# From Plant-Derived Compound to Bioactive Agent in Dentistry: The Expanding Role of Thymol

**DOI:** 10.3390/dj14090604

**Published:** 2026-09-18

**Authors:** Mhd Kher Alsaeyd Ahmad, Ștefania Dinu, Ștefania-Irina Dumitrel, Ramona Amina Popovici, Doina Chioran

**Affiliations:** 1 Doctoral School of Dental Medicine, “Victor Babeș” University of Medicine and Pharmacy, 9 Revolutiei 1989 Ave., 300070 Timisoara, Romania; mhd.alsaeyd-ahmad@umft.ro; 2 Department of Pedodontics, Faculty of Dental Medicine, “Victor Babeș” University of Medicine and Pharmacy, 9 Revolutiei 1989 Ave., 300041 Timisoara, Romania; 3 Pediatric Dentistry Research Center, Faculty of Dental Medicine, “Victor Babeș” University of Medicine and Pharmacy, 9 No., Revolutiei Bv., 300041 Timisoara, Romania; 4 Research Centre for Pharmaco-Toxicological Evaluations, “Victor Babeș” University of Medicine and Pharmacy, 2 Eftimie Murgu Square, 300041 Timisoara, Romania; 5 Department of Toxicology, Drug Industry, Management and Legislation, Faculty of Pharmacy, “Victor Babeș” University of Medicine and Pharmacy, Eftimie Murgu Sq. No. 2, 300041 Timisoara, Romania; 6 Department of Management and Communication in Dental Medicine, Faculty of Dental Medicine, “Victor Babeș” University of Medicine and Pharmacy, 300041 Timisoara, Romania; ramona.popovici@umft.ro; 7 Department of Anesthesiology and Oral Surgery, “Victor Babes” University of Medicine and Pharmacy, Eftimie Murgu Sq. No. 2, 300041 Timisoara, Romania; chioran.doina@umft.ro; 8 Research Center of Dento-Alveolar Surgery, Anesthesia and Sedation in Dental Medicine, “Victor Babeș” University of Medicine and Pharmacy, Eftimie Murgu Sq. No. 2, 300041 Timisoara, Romania

**Keywords:** thymol, dentistry, preventive dentistry, biofilms, prosthodontics, endodontics, periodontal diseases, dental materials, drug delivery systems

## Abstract

**Background:** Thymol has been considered a possible bioactive phytocompound in dentistry due to its antimicrobial, antibiofilm, antifungal and anti-inflammatory properties. The current narrative review aims to critically review the present evidence on biological properties, mechanism of action, formulations and applications of thymol in preventive dentistry, periodontics, endodontics, prosthodontics and emerging dental materials and delivery systems, highlighting the current limitations and future perspectives. **Methods:** A literature search was carried out on PubMed, Scopus, ScienceDirect and Google Scholar databases. The search included publications related to thymol and its applications in oral health and dentistry. **Results:** The most recent evidence supports the adjunctive use of thymol in preventive and periodontal oral-care products, particularly in essential oil-based formulations. Moreover, recent advances in nanoformulations and dental materials suggest that its therapeutic potential can be further improved by optimized delivery systems. **Conclusions:** Thymol is a naturally occurring active compound that offers numerous benefits in various dental applications due to its multifaceted properties and safety profile. Preliminary and clinical evidence is encouraging, but more standardized clinical studies are needed to better define its long-term efficacy, optimal formulations, therapeutic concentrations, and clinical applicability in dentistry.

## 1. Introduction

As the WHO states, “oral health is a key indicator of overall health, well-being, and quality of life” [1]. However, oral care is often overlooked, affecting nearly 4 billion people worldwide. The most frequently encountered dental problems are plaque buildup and gingivitis, which, if left untreated, can lead to tooth loss and systemic diseases such as cardiovascular disease, diabetes, and Alzheimer’s [2,3]. Other dental conditions, such as periodontal disease, endodontic infections, denture-associated candidiasis, and peri-implant biofilm-related complications, also pose major challenges in dentistry, as they are associated with microbial adhesion, biofilm maturation, and persistent host inflammatory responses [4,5,6,7]. Biofilms are not simply accumulations of microbes, but rather complex communities composed of multiple coaggregated microbes, which can increase resistance to antimicrobial agents and reduce the effectiveness of conventional therapies [6,8]. Moreover, the cross-kingdom interaction between different species of microorganisms enhances biofilm formation (e.g., between *Streptococcus mutans* and *Candida albicans*) and further amplifies virulence and disease progression. Current treatments include synthetic antimicrobials such as chlorhexidine and hydrogen peroxide, which have been shown to be insufficient to destroy infectious organisms beyond the well-formed matrix material [4]. Additionally, chlorhexidine, which is currently the most widely used antiseptic, leads to resistance and causes many adverse effects (tooth discoloration, taste disturbances, allergic reactions) [9,10].

To overcome these limitations, natural alternatives are often preferred. This category includes thymol, the main compound in *Thymus vulgaris* (thyme), as described in the European Pharmacopoeia. This compound possesses numerous properties, including antibacterial, antifungal, antioxidant, and anticancer effects [11]. Due to these biological properties, thymol has been incorporated into several oral-care products, particularly essential oil-based mouthwashes, varnishes, and toothpaste [12]. Despite the increasing curiosity in thymol-containing oral-care products and a large number of experimental studies, the available evidence is fragmented across different dental fields and formulations [4,13,14,15]. Most studies focus on specific antimicrobial or antibiofilm effects, often in combination with other active compounds, which makes it difficult to evaluate the independent therapeutic contribution [15,16]. Furthermore, advances in nanotechnology and dental biomaterials have expanded the potential applications of thymol beyond conventional mouthwashes and varnishes, although these methods are still mostly at the preclinical stage [13,17,18,19]. Hence, the novelty of the present review is that it provides a perspective on the applications of thymol in various dental fields, including both conventional oral-care applications and novel nanoformulation and biomaterial-based approaches. In addition, this paper critically assesses the current level of evidence, limitations, and translational challenges associated with the use of thymol in dentistry.

Taking these points into consideration, the present narrative review aims to provide a synthesis and critical discussion on the current evidence on biological properties, mechanisms of action, formulations, and applications of thymol in preventive dentistry, periodontics, endodontics, prosthodontics, dental materials and delivery systems, as well as to highlight current limitations and future perspectives.

## 2. Materials and Methods

This study was conducted as a narrative review. It focused on identifying the biological properties, mechanisms of action, and oral applications of thymol in dentistry. The literature search was conducted using PubMed and Scopus as the principal bibliographic databases, while ScienceDirect and Google Scholar were used as supplementary sources to identify additional relevant publications. The last searches were conducted for PubMed on 17 May 2026, Scopus on 10 May 2026, ScienceDirect on 28 April 2026, and Google Scholar on 20 April 2026. The search included articles published in English related to thymol and its applications in dentistry. The keywords most frequently used, either alone or in combination, were: “thymol”, “dentistry”, “oral biofilm”, “periodontal disease”, “gingivitis,” “endodontics,” “*Candida albicans*,” “*Enterococcus faecalis*,” “oral care products,” “mouthwash,” “prosthodontics,” “dental materials,” “nanoformulations,” and “drug delivery systems”.

For PubMed, the search strategy combined MeSH terms with free-text terms in the title and abstract, as follows:

(“Thymol”[MeSH Terms] OR thymol[Title/Abstract]) AND (“Dentistry”[MeSH Terms] OR “Preventive Dentistry”[MeSH Terms] OR “Biofilms”[MeSH Terms] OR “Prosthodontics”[MeSH Terms] OR “Endodontics”[MeSH Terms] OR “Periodontal Diseases”[MeSH Terms] OR “Dental Materials”[MeSH Terms] OR “Drug Delivery Systems”[MeSH Terms] OR dentistry[Title/Abstract] OR dental[Title/Abstract] OR oral[Title/Abstract] OR biofilm*[Title/Abstract] OR periodontal[Title/Abstract] OR gingivitis[Title/Abstract] OR endodontic*[Title/Abstract] OR prosthodontic*[Title/Abstract] OR mouthwash*[Title/Abstract] OR “oral care”[Title/Abstract] OR nanoformulation*[Title/Abstract] OR “*Candida albicans*”[Title/Abstract] OR “*Enterococcus faecalis*”[Title/Abstract]) AND English[Language].

For Scopus, the corresponding search was performed in the title, abstract, and keyword fields using the following strategy:

TITLE-ABS-KEY (thymol AND (dentistry OR dental OR oral OR “preventive dentistry” OR biofilm* OR “periodontal disease*” OR gingivitis OR endodontic* OR prosthodontic* OR “dental material*” OR mouthwash* OR “oral care” OR nanoformulation* OR “drug delivery system*” OR “*Candida albicans*” OR “*Enterococcus faecalis*”)).

ScienceDirect and Google Scholar were searched using combinations of the same core concepts and were used primarily to supplement the database searches and identify relevant publications that might not have been retrieved through the principal searches.

The inclusion criteria were: (1) publications addressing thymol or thymol-containing formulations in relation to dentistry or oral health; (2) studies investigating the biological properties, mechanisms of action, antimicrobial or antibiofilm effects, formulations, or dental applications of thymol; (3) original research articles, clinical studies, systematic reviews, and narrative reviews; (4) articles published in English; and (5) studies published predominantly from 2020 onward, with earlier studies included when considered relevant for foundational evidence or historical context.

The exclusion criteria were: (1) publications unrelated to dentistry or oral health and without relevance to the biological or mechanistic aspects of thymol discussed in this review; (2) conference abstracts and other publications providing insufficient information for assessment; (3) duplicate records; and (4) publications that, after title, abstract, or full-text assessment, did not contribute relevant evidence to the objectives of the review.

Article selection was based on relevance to the scope and objectives of the review and on the contribution of each publication to understanding the biological properties, mechanisms, formulations, and dental applications of thymol. No formal risk-of-bias or methodological-quality assessment tool was used because of the review’s narrative design and the variety of the included evidence. Rather, when evaluating the strength and clinical significance of the evidence, factors such as study design, experimental features, sample size when appropriate, reported results, and methodological constraints were taken into account. The searches identified 1041 records across the two principal databases (PubMed, *n* = 454; Scopus, *n* = 587). After removal of 318 duplicate records, 723 unique records remained and were screened on the basis of their titles and abstracts. At this stage, records that were clearly unrelated to thymol, dentistry, or oral health were excluded. Following title and abstract screening, 176 articles were assessed in full text for eligibility. Full-text publications were excluded if they did not address the biological properties, mechanisms of action, formulations, or applications of thymol relevant to dentistry or oral health, or if insufficient information was available to determine their relevance. A total of 112 publications were included in the final narrative synthesis. Supplementary searches of ScienceDirect and Google Scholar, together with reference-list screening of relevant publications, were used to identify additional supporting literature where appropriate.

## 3. Chemical Structure and Properties

**Figure 1:**
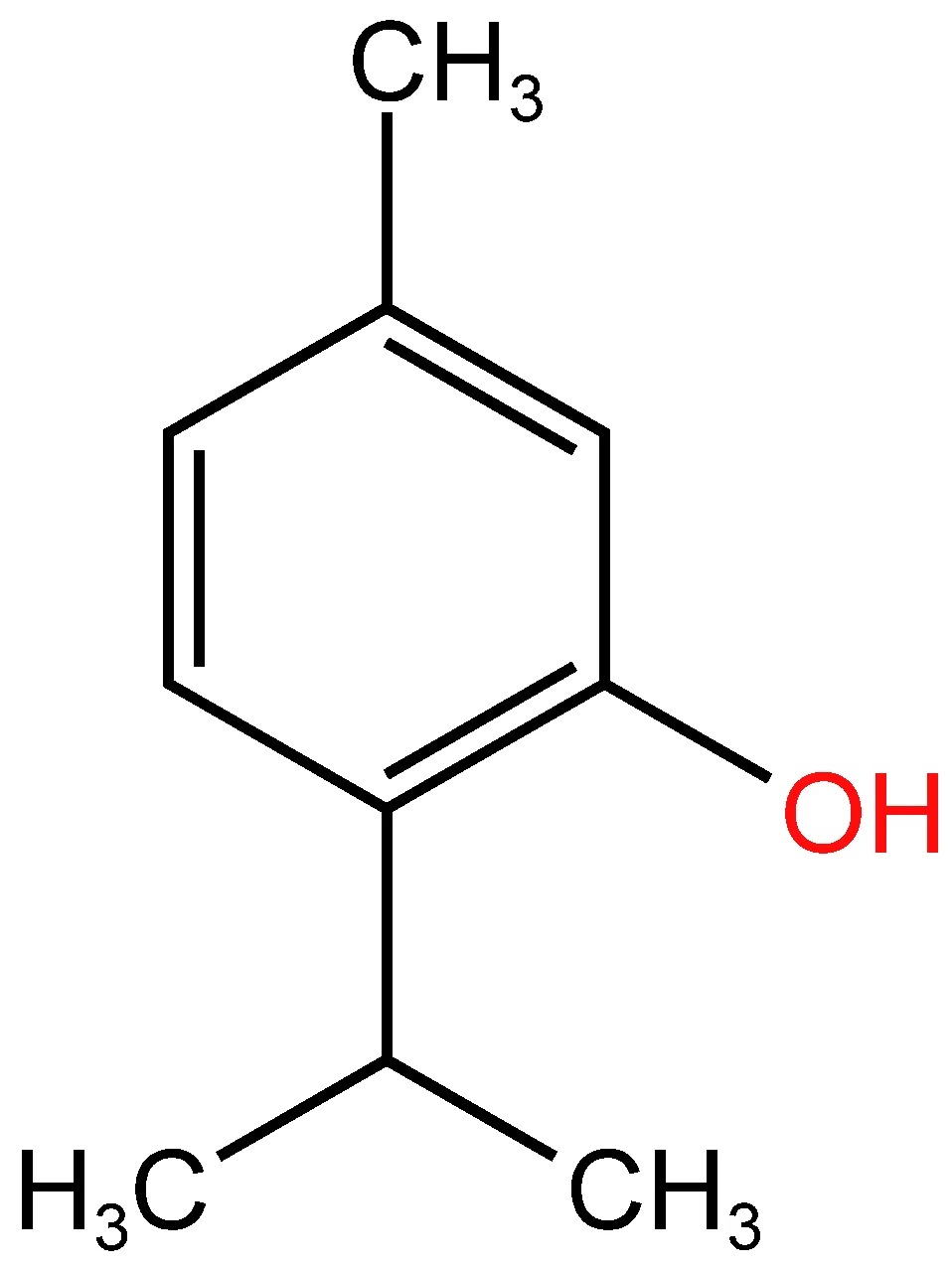
The chemical structure of thymol. Created with KingDraw.com (Accessed on 16 April 2026 https://www.kingdraw.com/indexen?name=download).

Thymol (2-isopropyl-5-methylphenol; C_10_H_14_O) is a monoterpene, which is naturally found in *Thymus vulgaris* [20,21]. It possesses a phenolic hydroxyl group and an isopropyl substituent (Figure 1), which confer its chemical and biological activity [21,22].

Thymol is a volatile compound that has a characteristic pungent odor, being slightly soluble in water but highly soluble in polar and nonpolar solvents. It is stable when stored in a cool place, protected from light, and in airtight containers; however, because of its potential reactivity, thymol is incompatible with strong acids, alkalis, and oxidizing agents [20,23].

The hydroxyl group is responsible for its antioxidant activity through its ability to neutralize free radicals via dehydrogenation. In contrast, the isopropyl group increases lipid solubility, facilitating penetration through biological membranes, disrupting their integrity, and impairing cellular structure and function [4,21,22,23]. These physicochemical properties contribute to the antiseptic activity and justify its inclusion in various pharmaceutical products used in dentistry.

After oral administration, thymol is rapidly absorbed and gradually eliminated within approximately 24 h. Its oral bioavailability, primarily in the form of thymol sulfate, is relatively low (approximately 16%), while the plasma half-life is around 1.5 h [11]. These pharmacokinetic characteristics may influence its retention and therapeutic performance in the oral cavity and partly explain the rising interest in sustained-release formulations and nanoformulation-based delivery systems for dental applications [11,13,19].

## 4. Biological Properties and Mechanisms of Action in the Oral Environment

### 4.1. Antimicrobial and Antibiofilm Activity

**Figure 2:**
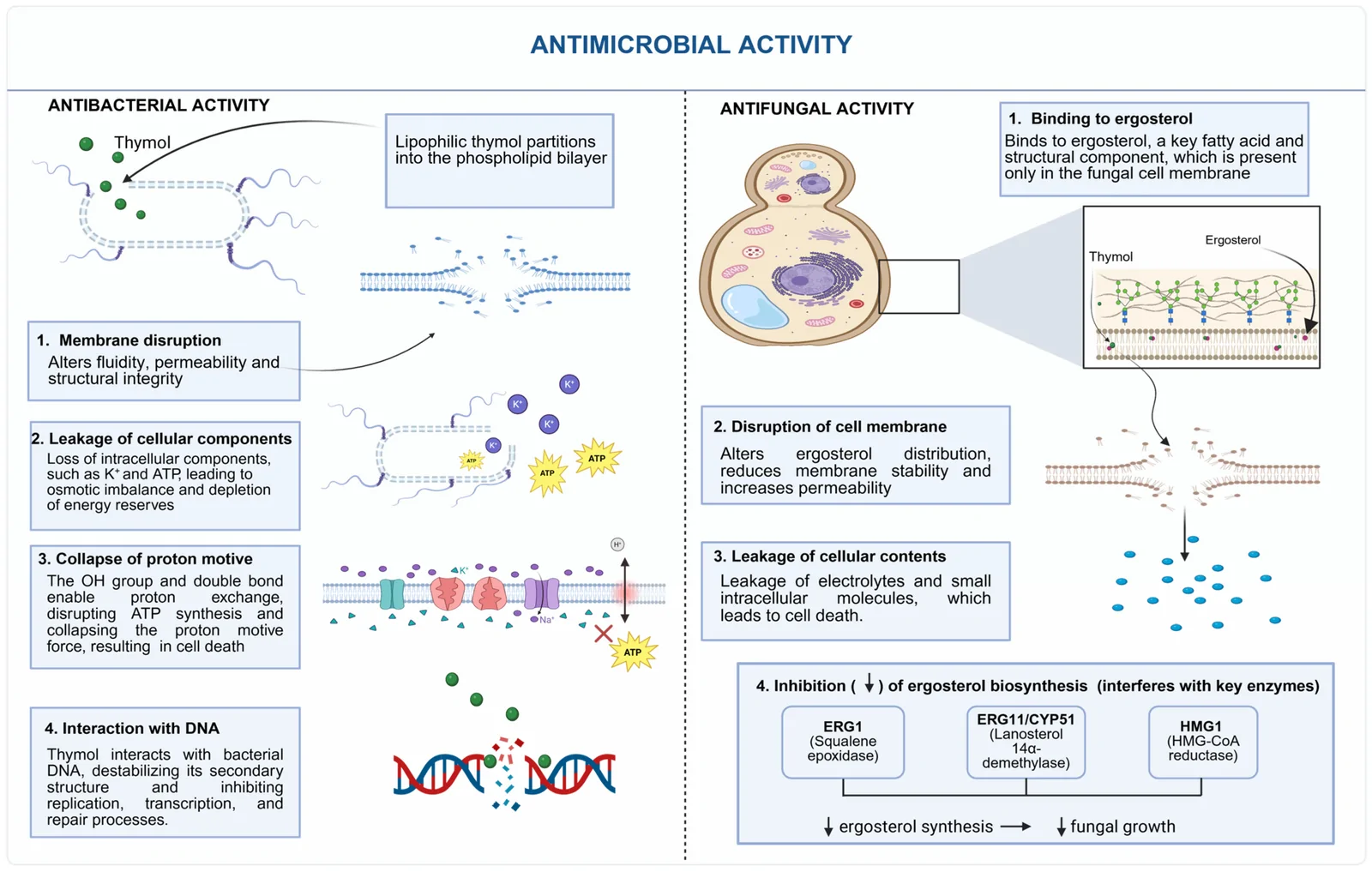
Schematic representation of thymol’s antimicrobial activity. Image created with Biorender.com (accessed on 7 May 2026; https://www.biorender.com/).

Thymol has a broad-spectrum activity, acting against Gram-positive and Gram-negative bacteria and fungi. It has inhibitory and bactericidal effects on plenty of microorganisms, such as *Staphylococcus aureus*, *Escherichia coli*, *Salmonella* spp., *Streptococcus* sp., *Klebsiella pneumoniae*, and *Mycobacterium tuberculosis* [23,24,25,26]. The antibacterial activity is attributed to the ability to rupture bacterial cell membranes. Because it is a fat-loving compound, it penetrates the phospholipid bilayer (Figure 2), thereby altering the fluidity, permeability, and structural integrity of the cell membrane. As a result, intracellular components such as potassium ions (K^+^) and adenosine triphosphate (ATP) leak out, causing osmotic imbalances and depletion of cellular energy reserves [23,27]. Because of the OH group and the double bond, it acts as a proton exchanger, disrupting ATP synthesis and causing the proton motive force to collapse, resulting in cell death [23]. Moreover, thymol interacts with bacterial DNA, destabilizing its secondary structure and affecting replication, transcription, and repair processes [28]. Other mechanisms described in the literature imply that it impairs cellular energy metabolism, membrane transport, nutrient uptake, ionic homeostasis, and bacterial cell division [25,29].

Multiple studies have shown that thymol has antifungal activity against various species, including *Candida* spp., *Aspergillus* spp., *Fusarium* spp., and *Mucor* spp. [23,30,31], because it binds to ergosterol, a key fatty acid and structural component, which is present only in the fungal cell membrane [32]. This interaction alters ergosterol distribution, reduces membrane stability, and decreases the barrier’s permeability. Consequently, membrane permeability increases, causing the leakage of electrolytes and small intracellular molecules until cell death occurs [23,33,34]. In addition, thymol interferes with ergosterol biosynthesis through various signaling pathways such as ERG1, ERG11/CYP51, and HMG1, which inhibit fungal growth [30,31].

**Figure 3:**
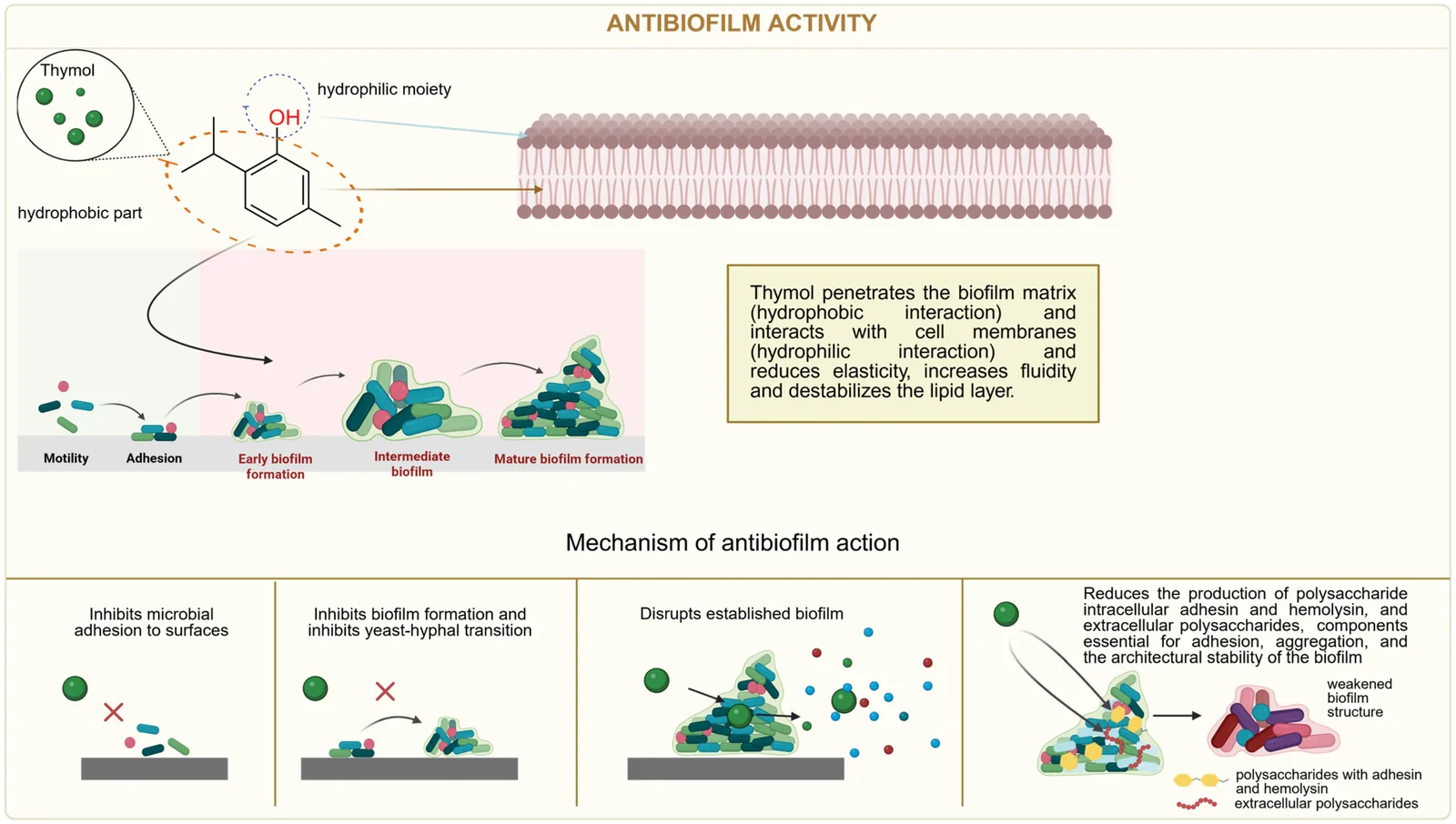
Schematic representation of thymol’s antibiofilm activity. Image created with Biorender.com (accessed on 7 May 2026; https://www.biorender.com/).

Beyond its activity against planktonic microorganisms, thymol inhibits and eradicates bacterial biofilms (Figure 3), which are essentially aggregates of microorganisms in which cells adhere to one another and also to a surface [24,25,26,27,34]. Compared to planktonic cells, biofilms have increased resistance to antimicrobial agents and possess defense mechanisms that can remain dormant under unfavorable conditions, making them particularly difficult to eliminate with conventional therapies [35].

Thymol’s hydrophilic moiety is attracted to the polar side of the membrane. Conversely, the hydrophobic benzene ring and its lipophilic chains penetrate the inner layers of the biofilm. This interplay affects the integrity of the biofilm because it impairs membrane structure by reducing its elasticity, increasing fluidity, and destabilizing the lipid layer [26].

It also inhibits biofilm formation by decreasing microbial adhesion to the surface. Microscopic analyses revealed reduced adhesion even at sub-inhibitory concentrations in the case of *Candida albicans* and *Streptococcus mutans* [4,36,37]. In systems containing either one or two species, thymol prevented biofilm formation and inhibited the yeast-hyphal transition [4]. In the case of polymicrobial biofilm formation, thymol inhibits biofilm formation in the intermediate phase and destroys biofilms already formed in the intermediate and mature phases [35]. It is effective even against resistant bacteria such as MRSA (methicillin-resistant *Staphylococcus aureus*), as well as against *Streptococcus mutans* and *Lactobacillus acidophilus*, especially when used in combination therapy with other therapeutic agents [35,36]. Furthermore, it reduces the production of polysaccharide intracellular adhesin and hemolysin, and extracellular polysaccharides, components essential for adhesion, aggregation, and the architectural stability of the biofilm [36].

### 4.2. Antioxidant and Anti-Inflammatory

**Figure 4:**
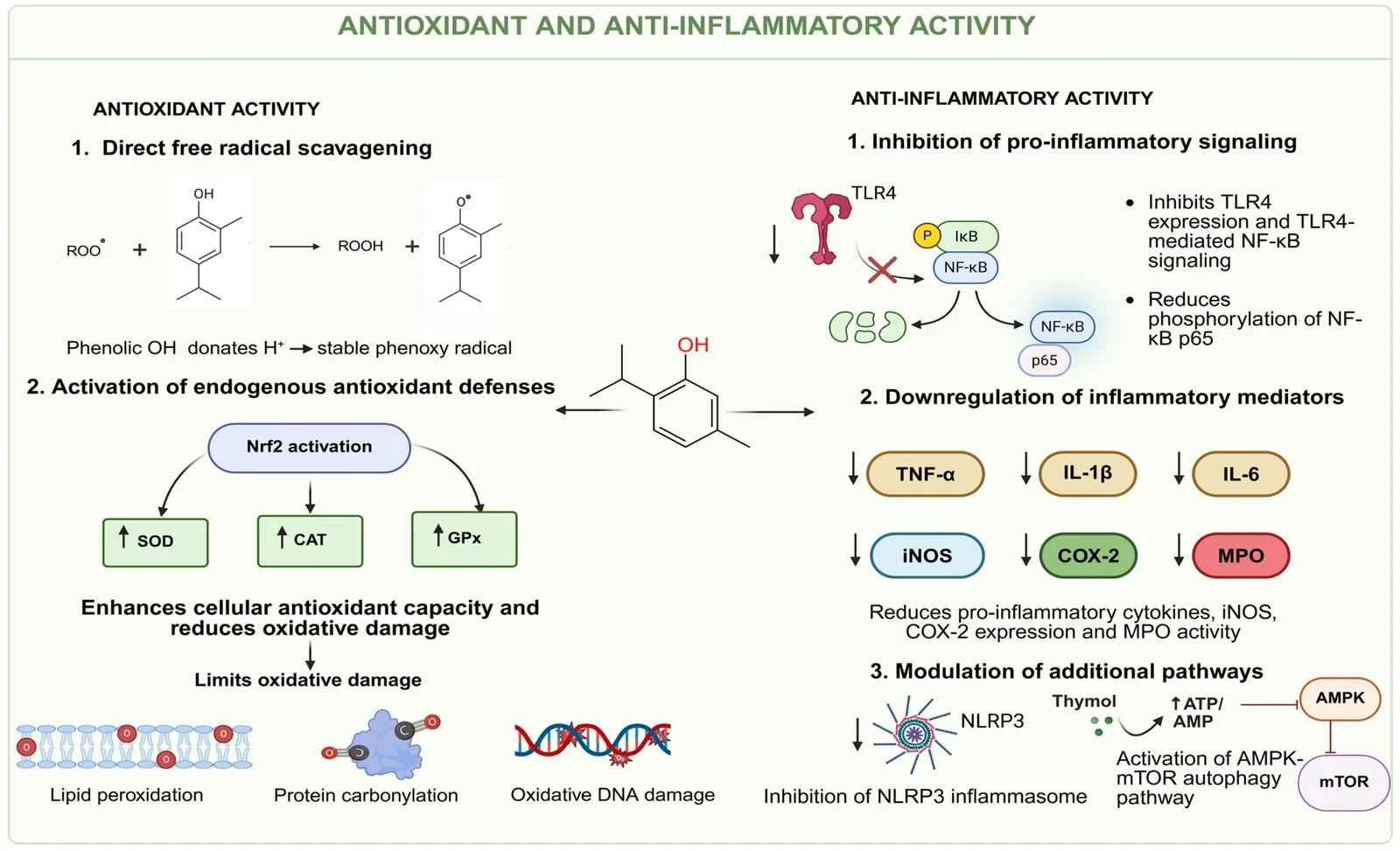
Schematic representation of thymol’s antioxidant and anti-inflammatory activity. Image created with Biorender.com (accessed on 8 May 2026; https://www.biorender.com/).

The anti-inflammatory and antioxidant properties of thymol are especially important in the field of dentistry, as oxidative stress and inflammation contribute to tissue damage and the development and onset of diseases [13,36]. Its antioxidant potential stems from the ability to neutralize free radicals and limit lipid peroxidation, protein carbonylation, and oxidative DNA damage, which exacerbate cellular dysfunction and trigger inflammatory processes (Figure 4) [23]. Free radicals are neutralized by the phenolic hydroxyl group, which, by donating hydrogen atoms, forms relatively stable phenoxy radicals [23,24]. Furthermore, it strengthens cellular antioxidant mechanisms by modulating the expression of key enzymes, including superoxide dismutase (SOD), catalase (CAT), and glutathione peroxidase (GPx), primarily through activation of the Nrf2 signaling pathway [38]. Concurrently, thymol lowers the expression of pro-inflammatory cytokines (TNF-α, IL-1β, and IL-6) while regulating signaling pathways implicated in inflammatory response. More specifically, it suppresses NF-κB signaling, including the phosphorylation of NF-κB p65, and inhibits TLR4 expression and TLR4-mediated NF-κB signaling [38,39]. Other anti-inflammatory effects are suppressed through the reduction in iNOS and COX-2 expression, as well as MPO activity, which limits inflammatory cells from infiltrating and damaging the tissue [38]. In addition, thymol can suppress NLRP3 inflammasome activation and regulate the AMPK–mTOR–autophagy signaling pathway, which attenuates inflammatory processes and protects against cellular injury [23].

### 4.3. Antitumoral

**Figure 5:**
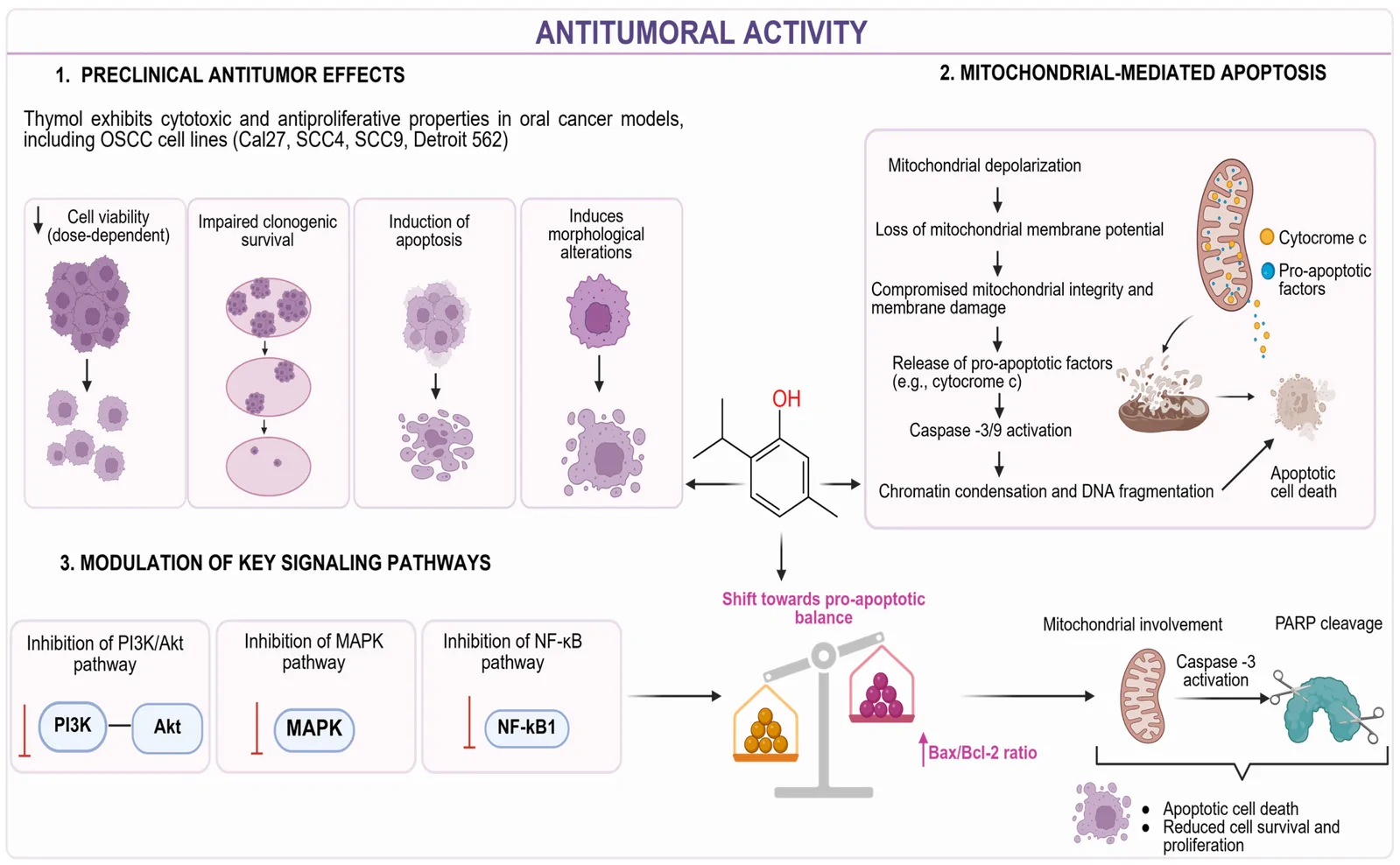
Schematic representation of thymol’s antitumoral activity. Image created with Biorender.com (accessed on 9 May 2026; https://www.biorender.com/).

Thymol has gained growing interest in recent years for its putative antitumor properties, specifically concerning oral squamous cell carcinoma and other malignant neoplasms of the head and neck (Figure 5) [12,40,41,42]. Nevertheless, existing data are confined to preclinical investigations. Thymol exhibited cytotoxic and antiproliferative properties in various oral cancer models, including oral squamous cell carcinoma cell lines such as Cal27, SCC4, and SCC9 [43]. It demonstrated dose-dependent cytotoxicity in a pharyngeal carcinoma model; however, this effect was only moderately selective, as normal cells were similarly affected at elevated concentrations. Thymol diminished cancer cell viability, impaired clonogenic survival, and induced morphological alterations indicative of apoptosis in oral and oropharyngeal systems [12,44]. From a mechanistic standpoint, the most consistently validated effect is mitochondria-mediated apoptosis, evidenced by mitochondrial depolarization, loss of mitochondrial integrity, chromatin condensation, membrane damage, cytochrome c release, and caspase-3/9 activation and a dose-dependent increase in apoptotic cell death [12,40]. More detailed mechanistic studies indicate that these effects may be associated with the modulation of signaling pathways related to cell survival and proliferation, such as PI3K/Akt, MAPK, and NF-κB, in addition to a transition towards a pro-apoptotic balance, marked by increased BAX/Bcl-2 ratio and activation of mitochondrial-mediated apoptotic pathways involving caspase-3 and PARP cleavage [40,45]. These findings indicate that thymol is not yet an established anticancer agent in dentistry, but rather a promising bioactive oral compound whose antitumor potential requires further investigation, particularly with regard to dose optimization, formulation, and long-term safety in normal oral tissues.

## 5. Thymol in Oral Care Products

Thymol is most commonly found in mouthwashes that are blended with essential oils and other active ingredients [46]. Currently, mouthwashes [15,46,47] and, to a lesser extent, toothpastes [12,48] and varnishes [49] are the main products that contain thymol and are found on the market. Thymol is present at a concentration of 0.064% along with eucalyptol (0.092%), menthol (0.042%), and salicylate (0.060%) within the fixed essential oil formulation that is most frequently discussed in professional literature on oral health [15]. Apart from these formulations, thymol appears in chlorhexidine–thymol varnishes used for professional plaque and bacterial control, in which both thymol and chlorhexidine are present at 1% (*w*/*w*) [49,50]. Furthermore, it has been incorporated into various oral gels containing thymol and nano-encapsulated systems, but these are described primarily in pilot or experimental studies, suggesting that these formats remain less standardized and less clinically established than mouthwashes [24,51].

## 6. Applications in Dentistry

### 6.1. Preventive Dentistry

Thymol is most commonly found in preventive dental care products, such as mouthwashes containing essential oils, which have been evaluated in both short- and long-term clinical settings, as they have been in use for many years in daily oral care as adjuncts to mechanical plaque control [24,46]. Mouthwashes containing thymol have been shown to reduce supragingival plaque accumulation, inflammation in the gingival area, and halitosis [24,46], due to their capacity to penetrate the biofilm, disrupt bacterial membranes and enzymatic activity, and interfere with bacterial aggregation and plaque maturation [24,35]. One advantage of mouthwashes, in addition to toothbrushing, is that they can infiltrate areas that are difficult to reach mechanically, such as the interproximal spaces. Long-term use has shown a good safety profile, as they are generally well-tolerated [23]. Furthermore, there has been no evidence that they induce undesirable changes in the supragingival microbiota or reduce sensitivity to antiseptics over time [4,23,52]. Both alcohol-containing and alcohol-free mouthwashes have shown that there is no loss of therapeutic effect in alcohol-free mouthwashes, which may improve long-term patient compliance [53].

Toothpastes with thymol have been shown to reduce volatile sulfur compound (VSC) levels, which are strongly linked to halitosis. Moreover, this effect was accompanied by inhibitory activity against malodor-associated bacteria, including *Porphyromonas gingivalis* and *Fusobacterium periodonticum*, indicating a potential role in modulating the oral microbial ecosystem [48]. Kashi et al. have suggested that sugar-free chewing gum could represent an innovative way to deliver thymol in preventive oral care [54], as medicated chewing gum raises plaque pH, boosts salivary flow, and supports remineralization, all while providing some mechanical cleaning. This approach may be especially useful for children, since they do not always follow normal oral hygiene rules [54,55,56]. On top of that, this strategy seems viable, especially because it could lead to longer-lasting antimicrobial activity and persistence in the oral cavity. However, more research is needed to see if it is feasible, effective, and useful in clinical applications.

Thymol contributes to caries prevention by inhibiting key virulence factors of *Streptococcus mutans*, including adhesion, biofilm formation, and acid production [54,56]. In a biofilm made up of two types of microbes (*Streptococcus mutans* and *Candida albicans*), thymol successfully reduced microbial load, but more importantly, it changed the biofilm’s structure and behavior [4]. Chlorhexidine–thymol varnishes have been associated with reduced levels of *Streptococcus mutans* and, in certain situations, with improved outcomes in the advancement of root caries [52,55,57].

Some of the applications of thymol in preventive dentistry are summarized in Table 1.

The evidence supporting thymol in preventive dentistry varies substantially according to the formulation and proposed application. Randomized clinical trials provide the strongest direct clinical evidence, particularly for thymol-containing mouthwashes, demonstrating reductions in plaque indices, gingival inflammation, and oral malodor, although thymol is generally administered as part of a multicomponent formulation [58,60]. Chlorhexidine–thymol varnishes are also supported by randomized clinical studies, with evidence of reductions in cariogenic bacterial counts and, in selected populations, reduced development or progression of root caries [50,61]. However, the clinical findings are not entirely consistent, as limited effects on plaque and gingivitis have also been reported with some varnish protocols [50]. Further clinical evidence is provided by an in vivo study of chlorhexidine–thymol varnish, which reports improvements in gingival parameters and reductions in *S. mutans* and *P. gingivalis* levels [59]. However, in vivo experimental evidence is limited, although a *Galleria mellonella* model showed improved survival and reduced microbial burden after thymol treatment [4]. Most of the studies evaluating the independent preventive activity of thymol have been performed in vitro. They have revealed antibacterial, antifungal, antibiofilm, and antivirulence activities toward cariogenic microorganisms [4,35,52,59,62,63,64,65,66]. Although thymol-containing toothpaste has shown promising antimicrobial and anti-halitosis activity, the evidence presented here is laboratory-based [48]. Likewise, thymol delivery through chewing gum remains a proposed preventive strategy without direct clinical validation and should currently be regarded as investigational [54,55,56]. Overall, thymol has the most established clinical role as an adjunctive component of mouthwashes and selected varnish formulations, whereas its use as an isolated preventive agent, in toothpaste-specific therapeutic applications, or in novel delivery systems such as chewing gum requires further controlled clinical investigation.

### 6.2. Periodontal Disease

Thymol’s activity has been extensively studied in periodontal diseases (e.g., gingivitis and periodontitis), because of its combined antimicrobial, anti-inflammatory, and antioxidant properties [50,67,68]. Periodontal diseases are initiated by dysbiotic biofilms and sustained by a dysregulated host inflammatory response. Thymol acts against periodontal disease-linked pathogens, such as *Porphyromonas gingivalis* and *Aggregatibacter actinomycetemcomitans* [68,69] by disrupting bacterial cell membranes and causing leakage of intracellular components [70]. Moreover, it interferes with the enzymatic systems involved in bacterial survival and biofilm maturation, reducing bacterial viability, disrupting aggregation, and destabilizing biofilms [35]. Thymol can attenuate bacterial toxicity on *Porphyromonas gingivalis* and significantly reduce biofilm formation. Jia et al. developed an LPS-stimulated rat gingival fibroblast model of periodontitis and showed that thymol (10–40 μg/mL) reduced pro-inflammatory cytokines (IL-1β, IL-6, TNF-α) while increasing anti-inflammatory cytokine expression (IL-10). In the same context, thymol raised the ratio of osteoprotegerin (OPG) to receptor activator of nuclear factor-κB ligand (RANKL) and inhibited the phosphorylation of NF-κB p65 and IκBα, suggesting a modulatory effect on inflammatory signaling pathways and a potential role in decreasing osteoclastogenesis [71].

The destruction of periodontal tissue does not depend exclusively on the presence of pathogenic microorganisms, but also on the magnitude and persistence of the host’s immune response [72]. Thymol helps limit tissue damage and disease progression by providing an additive effect that modulates both microbial load and inflammatory signaling, such as NF-κB-related signaling pathways, suppression of osteoclastogenesis, and modulation of the RANKL/OPG axis [73]. Clinical and translational evidence further supports these observations. Products that are designed for oral care, which contain thymol (usually as part of essential oil formulations), reduce bacterial plaque accumulation, gingival inflammation, and bleeding indices when used as adjuncts to mechanical plaque control [15,50,58,74].

A great advantage is that oral care thymol formulations are generally well-tolerated. They cause fewer adverse effects compared to conventional antiseptics, such as chlorhexidine, which are associated with tooth discoloration and changes in taste with long-term use. Furthermore, current data suggest that thymol does not disrupt the overall balance of the oral flora, but rather exerts a selective effect on pathogenic species [24,67].

Some of the applications of thymol in periodontal disease are summarized in Table 2.

Evidence for periodontal applications of thymol spans both clinical and preclinical levels. The highest level of evidence is provided by systematic reviews of thymol-containing essential-oil mouthrinses [15,74], supported by several randomized controlled trials [50,53,57,58,75]. More limited clinical evidence is available from a small pilot study evaluating a thymol–carvacrol gel [76]. Preclinical evidence includes in vitro studies [35,68,69,71] and combined in vitro and in vivo experimental research [73], whereas no ex vivo studies were identified among the principal studies summarized in this section. Overall, the evidence supports thymol-containing essential-oil mouthrinses as adjuncts to mechanical plaque control, particularly for plaque and gingival inflammation. Other proposed periodontal applications, including locally delivered thymol formulations and their potential effects on periodontal inflammation and bone loss, remain primarily experimental and require further clinical validation.

### 6.3. Endodontics

Evidence about the efficacy of thymol in endodontics is sparse, and most of it comes from in vitro studies. Recently, Manuel et al. evaluated the antibiofilm activity of liquid thymol and thymol vapor on early biofilms of *Enterococcus faecalis*, *Streptococcus mutans*, and *Aggregatibacter actinomycetemcomitans*. The tests were undertaken in a curved-canal resin block model, which was designed to simulate root canal conditions. In planktonic culture, thymol had minimum inhibitory and bactericidal concentrations of approximately 0.8–1.0 mg/mL. Liquid thymol (10–100 mg/mL) and thymol vapor (5.0 mg/mL) significantly lowered bacterial viability in 3-day biofilm models. Mechanical instrumentation combined with thymol vapor at 1.0 mg/mL improved the removal of early biofilm (in particular *E. faecalis*). In contrast to direct exposure to liquid thymol and chlorhexidine, which caused marked cytotoxicity, 1.0 mg/mL thymol vapor was associated with lower cytotoxicity and did not significantly increase pro-inflammatory cytokine gene expression (IL-1β, TNF-α, IL-6) in L-929 fibroblasts [77]. These results suggest that thymol, especially in vapor form, may have antimicrobial benefits with a less toxic profile in endodontic applications. A commercial product, Cresophene^®^ (dexamethasone acetate + thymol), was compared with 20% chlorhexidine gel, 3% sodium hypochlorite, and saline for intracanal disinfection in 80 extracted single-rooted human teeth infected with *Enterococcus faecalis*. Cresophene^®^ diminished the bacterial load by 89.85% after 48 h of treatment, which was comparable to chlorhexidine (90.15%) and more effective than 3% sodium hypochlorite (75.57%) [78]. *E. faecalis* is strongly associated with persistent endodontic infections and failed root canal treatment due to its ability to survive nutrient deprivation, penetrate dentinal tubules (even more than 1000 μm), and create resistant biofilms [79,80]. Because of the aforementioned characteristics, *E. faecalis* is a major target in studies investigating alternative endodontic antimicrobial strategies [81,82]. In another study, *E. faecalis* strains isolated from infected root canals were exposed to pure thymol and *Lippia sidoides* essential oil at concentrations of 2.5% and 10% for 30 and 60 min. Both treatments significantly decreased colony-forming units within 72-h biofilms, with no statistically significant difference between the essential oil and thymol. Such evidence indicates that thymol is likely to be one of the major components responsible for the activity against *E. faecalis* biofilms [83].

Some of the applications of thymol in endodontics are summarized in Table 3.

The evidence for endodontic uses of thymol at present is limited to the lower preclinical levels of the evidence hierarchy. No systematic reviews or meta-analyses, randomized trials, non-randomized clinical studies or animal studies were identified to assess thymol’s endodontic efficacy within the studies described in this section. The available evidence is mostly based on in vitro studies including planktonic and biofilm models [77,83,84] and a model of intracanal infection using extracted human teeth [78]. These studies demonstrate antimicrobial and antibiofilm potential, especially against *E. faecalis*, but they are pre-clinical, not clinical evidence. Thus, thymol-based solutions, vapor formulations and thymol-containing intracanal products are still experimental for endodontic use and their routine clinical application needs to be supported by validation in controlled clinical studies.

### 6.4. Prosthodontics and Prosthesis-Associated Candidiasis

The colonization of removable dentures by microorganisms represents a major problem in dental prosthetics. The surface of dentures can facilitate microbial adhesion and biofilm accumulation due to their surface properties, the formation of a salivary film, and continuous exposure to the oral environment [85]. *Candida albicans* is frequently associated with denture stomatitis, as it adheres to the acrylic surface of dentures, thereby contributing to the development of this condition [7,85]. Some *Thymus* species, which contain approximately 60% thymol, have demonstrated activity against *Candida albicans*, with MIC_80_ values ranging from 0.0005 to 1.6690 μg/mL. The anti-yeast activity of thymol comes from its capacity to inhibit the transformation of *C. albicans* from yeast to hyphal form [86]. Other studies further support its suppressive effect on the filamentous development of *Candida albicans* and some related species, namely via hyphal-growth suppression, while also reducing fungal viability [87,88,89,90].

On two strains of *C. albicans*, thymol influenced the early stages of biofilm formation and mature biofilms. The metabolic activity of sessile cells was reduced by more than 90%, twice the MIC of planktonic cells [91].

Kaypetch et al. evaluated a new denture-cleaning product containing thymol on thermoset PMMA specimens with multi-species biofilms comprising *Streptococcus mutans*, *Streptococcus sanguinis*, *Staphylococcus aureus*, *Escherichia coli*, and *Candida albicans*. In time-dependent killing tests, the thymol-containing cleaning product exhibited more than 99.9% anti-*Candida* activity after 5 min and also demonstrated effective antimicrobial, antibiofilm, and stain-removing properties. The cleaning product did not adversely affect the physical properties of the acrylic material and cause toxicity due to residual substances leached from the resin, supporting the use of thymol-containing cleaning systems as a promising alternative for denture hygiene [92]. In a dynamic multispecies biofilm model developed on dental implant surfaces in the presence of *Candida albicans*, thymol suppressed filamentous growth during biofilm development. Although global biofilm biomass and viability were not significantly different in the thymol-treated condition, the viability of *Fusobacterium nucleatum* and *Porphyromonas gingivalis* was decreased, partially reversing the effect exerted by *C. albicans* on biofilm structure and vitality [14]. These findings suggest thymol’s potential role as a modulator of fungal–bacterial interactions on prosthetic surfaces.

Some of the applications of thymol in Prosthodontics and Prosthesis-Associated Candidiasis are summarized in Table 4.

Most evidence is from in vitro studies on antifungal and antibiofilm activity against *Candida* species, including biofilms formed on denture and implant surfaces [14,34,87,88,90,91,92,93,94]. In vivo experimental evidence is limited to a larval model for the toxicity assessment of an essential oil containing thymol [95], and no ex vivo studies were found. The available findings support the antifungal potential of thymol and its possible incorporation into denture-cleansing systems, but these applications are still at the preclinical stage. Clinical studies are needed to demonstrate that they are effective and safe and are compatible with prosthetic materials over the long term before they can be used as a matter of routine.

### 6.5. Dental Materials and Drug Delivery Systems

Thymol’s antimicrobial and anti-inflammatory effects have prompted research into its integration into dental materials and nanoformulations. Thymol nanoemulsions have demonstrated promising effects in oral infections, exhibiting long-lasting antibacterial activity, stability, and good dispersion in aqueous environments [13].

Topical antimicrobial products that are traditionally used have certain drawbacks that make them less effective over time, including insufficient penetration into mature biofilm layers, rapid elimination by saliva, and short retention time [96]. On this basis, modified release systems with thymol have been studied to solve some of these issues and constraints. These systems can improve penetration and bioavailability and reduce the number of administrations and systemic exposure [17,97,98]. These advantages may be particularly relevant for patients who require long-term oral care, especially those with reduced treatment adherence, such as children, older adults, and individuals with special needs.

Thymol-coated titanium surfaces displayed antibacterial activity against *Staphylococcus aureus*, along with good cytocompatibility in fibroblastic and pre-osteoblastic cells, and provided sustained release for up to 10 days [17]. In a related study, thymol-loaded microsponges in an in situ gel alleviated gingival inflammation, reduced tooth mobility, and prevented alveolar bone destruction. The high entrapment efficiency allowed the formulation to liberate thymol for about 10 h. In addition, it had good mucoadhesion and prolonged retention in periodontal tissues [99]. A methacrylate resin bonding system containing thymol exerted antibacterial activity against *Streptococcus mutans*, while maintaining good cytocompatibility. Bond strength and mechanical performance were comparable to those of conventional adhesives, suggesting thymol as a natural antibacterial additive for enamel bonding systems, with the potential to reduce secondary caries without compromising adhesive performance [97]. Zheng et al. reported that thymol-loaded UiO-66 metal–organic frameworks unveiled antimicrobial, anti-inflammatory, and osteogenic bioactivities, showing potential as multifunctional delivery systems for implant coatings and regenerative dental applications [100]. Together, these properties make thymol a strong candidate for preventing implant-associated infections and promoting successful osseointegration.

Nanoencapsulation is a well-established and thoroughly studied method because it optimizes the biological properties and stability of active molecules. In addition, it improves water solubility, which is a significant benefit for compounds like thymol, which is very poorly soluble in water [96,101]. Polymeric nanoparticles, especially those based on PLGA or chitosan, enhanced the water dispersibility of thymol, protected it from degradation, and provided sustained release in the oral cavity [19]. Furthermore, nanoencapsulated clove oil and thymol enhanced antimicrobial performance against cariogenic bacteria (*Streptococcus mutans* and *Streptococcus sobrinus*), having a persistent antimicrobial effect and better stability under oral conditions compared with free compounds [96].

Adding thymol to additively manufactured (3D printed) dental polymers could be a new research direction in the future. As 3D printed polymeric materials are increasingly being investigated for oral appliances, they offer an alternative to conventional and subtractive CAD-CAM materials. If such materials are functionalized with bioactive compounds such as thymol, they may have additional antimicrobial properties. However, the effects of adding thymol on the mechanical, biological, and long-term clinical performance of these materials need to be investigated separately [102].

Some of the applications of thymol in Dental Materials and Drug Delivery Systems are summarized in Table 5.

The documentation for thymol-containing dental materials and drug-delivery systems is largely preclinical [104,105]. A systematic review has evaluated thymol–chitosan delivery systems [19]; however, the evidence summarized for these applications is predominantly experimental rather than clinical. No randomized controlled trials evaluating these dental applications were identified among the studies summarized in this section. Limited in vivo evidence is available for selected nanoformulations and delivery systems [96,99,100], while ex vivo studies have investigated buccal permeability and drug delivery [13,106]. The majority of the remaining evidence is based on in vitro studies investigating antimicrobial activity, biocompatibility, material properties, and controlled release characteristics [17,32,97,102,103,104,105,106,107]. To summarize, nanoencapsulation and incorporation of thymol into dental materials present promising antimicrobial and drug-delivery properties, but these approaches are still mainly in the preclinical and translational stages. Further controlled clinical trials are needed to determine efficacy, safety and use in routine dental practice.

## 7. Safety

Thymol is considered a safe compound and has been classified by the U.S. FDA as “Generally Recognized as Safe” (GRAS). The EPA also assigns it a relatively low-risk profile under defined conditions of use [23]. Its safety profile has been further supported by the widespread use in oral care products (approximately 30% of mouthwashes contain thymol). Regulatory status, however, is only indicative of safety within known limits of exposure and does not represent a full pharmacological profile [12]. Thymol’s use in oral care products is still not sufficiently investigated in terms of therapeutic dosage, long-term safety, and cumulative exposure. There have been reports of cases in which excessive ingestion of mouthwashes containing thymol contributed to metabolic acidosis and systemic toxicity [108]. Also, concentrated thymol can irritate and corrode the skin in the area [109]. Nevertheless, most formulations have ethanol in them, which is thought to be the main cause of toxic reactions [110]. On the cellular level, thymol has shown concentration-dependent biocompatibility in human primary gingival fibroblasts, with acceptable cytocompatibility at lower concentrations but reduced viability at higher exposures [12]. These findings highlight that the oral biocompatibility of thymol is highly dependent on concentration and exposure conditions. Overall, thymol has an acceptable biosafety profile in current clinical settings, but further studies are needed to define long-term effects in the oral cavity.

## 8. Discussion

The data from the studies discussed in this review indicate that thymol has various applications in dentistry, given its multiple properties (antimicrobial, antibiofilm, antifungal, and anti-inflammatory). However, the clinical applicability of thymol appears to fluctuate depending on the dental field, formulation, concentration, delivery system, contact time, and whether or not it has been used in combination with other compounds.

The most common clinical formulations with thymol are mouthwashes and varnishes.

Despite the fact that thymol has demonstrated antimicrobial and antibiofilm activity in experimental studies [4,35,54,62], most clinical trials have evaluated it in combination with other active substances. Therefore, the extent to which thymol alone contributes to clinical efficacy remains difficult to determine. Based on the current evidence, thymol appears to play a role primarily as an adjunctive component rather than as a stand-alone therapeutic agent.

In periodontal diseases, the strongest clinical evidence supports thymol-containing essential-oil mouthwashes as adjuncts to mechanical oral hygiene [15,53,74]. The proof for other periodontal formulations is less consistent, limited by small sample sizes, multicomponent formulations and the difficulty of distinguishing the specific effect of thymol from other active ingredients [50,57,75,76].

In endodontic practice, there are far fewer studies, and most of them are in vitro. Although antimicrobial effects against *E. faecalis* and intracanal biofilms have been reported [77,78], the absence of adequate clinical evidence prevents thymol from currently being considered an established endodontic irrigant or intracanal medicament.

In prosthodontics, the antifungal and antibiofilm properties of thymol provide a rationale for its potential use in denture hygiene and denture-associated candidiasis [34,87,91,92]; however, the evidence remains predominantly preclinical. It is important to note that exposure time and concentration are critical factors that must be taken into account. For instance, longer immersion is needed for multispecies biofilm reduction, but higher concentrations of thymol-containing essential oil can alter PMMA roughness and color stability. Therefore, denture hygiene products containing thymol appear to be a promising strategy; however, their safety and material compatibility are highly dependent on optimal concentration and exposure protocols.

Experimental studies have demonstrated that thymol exhibits antinociceptive activity in animal pain models, suggesting that it may be involved in analgesic activity. A recent pilot study showed a significant reduction in pulpal pain after topical application of a multicomponent phytochemical formulation containing thymol. However, thymol was combined with several other bioactive compounds, making its independent contribution to the analgesic effect unclear. Thus, the potential role of thymol in the management of orofacial pain is still preliminary and requires specific clinical investigation [111,112].

As illustrated in the manuscript, topical treatments intended for the oral cavity have several disadvantages: poor penetration into the biofilm, rapid clearance via saliva, short retention time, and low water solubility. Precisely to overcome these issues, thymol has been incorporated into a variety of delivery systems such as nanoemulsions [13], chitosan- or PLGA-based nanoparticles [19], microsponges [99], metal–organic frameworks [100], resin-based systems [97], and titanium surface coatings [17]. Experimental proof validated that these systems significantly improved solubility and stability and provided sustained release, enhancing antibiofilm activity while maintaining biocompatibility. Furthermore, the addition of thymol to dental materials has been shown to prolong antimicrobial effects, with positive outcomes against secondary caries and peri-implant infections. In addition, some of these novel systems displayed anti-inflammatory effects and osteogenic potential [100], further supporting their relevance in periodontal therapy and regenerative applications. Despite these revolutionary formulations tackling and resolving some of the compound’s limitations, the results are preclinical, derived from in vitro studies or experimental models that do not fully replicate the complexity of the oral environment.

A major challenge in translating these experimental findings into clinical practice is whether antimicrobial concentrations of thymol that are effective in vitro can also be achieved and maintained safely in the oral cavity. The concentrations that were reported to be effective vary considerably depending on the microorganism, biofilm model, formulation, and exposure time, while data on the local pharmacokinetics and bioavailability of thymol in the oral environment remain insufficient. Salivary dilution and rapid clearance may further reduce local exposure, which limits direct extrapolation from static in vitro models to clinical conditions. Although sustained-release formulations have prolonged thymol release and retention in experimental settings [17,19,99], clinically relevant substantivity of thymol itself has not yet been adequately established. Furthermore, the extent to which interactions with salivary proteins modify the free and biologically active concentration of thymol remains scarcely characterized. Moreover, antimicrobial efficacy must be considered together with biocompatibility, since cytotoxicity is concentration- and exposure-dependent [12,77]. A similar balance is required for dental materials, as some thymol-containing formulations have maintained material properties, whereas higher concentrations or prolonged exposure may adversely affect surface roughness and color stability [92]. Therefore, antimicrobial activity alone is insufficient to establish clinical applicability, and future studies should define a therapeutic window that simultaneously provides effective antimicrobial activity, adequate oral retention, acceptable cytocompatibility, and preservation of dental material properties. In general, the present documentation concludes that thymol could play a multifunctional bioactive role in dentistry with encouraging applications in prevention, periodontics, prosthodontics, and materials. Nevertheless, the strength of proof varies widely between fields. The most direct clinical relevance is to mouthwash-based preventive and periodontal applications. Afterwards, prosthodontic applications have a strong antifungal rationale but are largely in vitro. Endodontic applications are preliminary. Finally, nanoformulations and dental materials are promising translational platforms that need further clinical validation. Thus, the most likely clinical role of thymol in the current scenario is as an adjunctive agent in optimized formulations rather than as a stand-alone therapeutic substitute. Though there is considerable evidence for thymol’s utility in preventive and periodontal care, its translation into predictable clinical outcomes has been poorly investigated. More research is needed focusing on clinically relevant endpoints such as incidence of caries, periodontal attachment gain, peri-implant tissue stability, success of endodontic treatment, and recurrence of denture-associated infections. Also, comparative investigations against standard treatments such as CHX, sodium hypochlorite, and conventional antifungal agents would help determine whether these advantages are meaningful in terms of efficacy, safety, compliance, and reduction in adverse effects.

## 9. Limitations

This study has some limitations. Across the studies, the level of evidence is heterogeneous, which affects both the strength and the generalizability of the conclusions. Studies with a higher level of evidence (systematic reviews and RCTs) are found in the sections on dental prevention and periodontal applications, while a large portion of the studies (in the endodontics, prosthodontics, and dental materials and nanoformulations section) are primarily derived from in vitro studies or other experimental models. Therefore, antimicrobial and antibiofilm effects cannot be translated to clinical conditions, where there are all kinds of host-microbe interactions, saliva, and other environmental factors. Another point is that thymol has often been tested in combination with other compounds, either as part of a multi-component product. Consequently, these factors make it more difficult to isolate the activity of thymol per se and create uncertainty regarding its mechanism of action—whether it is additive, synergistic, or due to the formulation itself. Another important point is that doses vary, being reported in μg/mL, mg/mL, % *v*/*v*, or dilution ratios, as do formulations, experimental models, and outcome measures. Furthermore, not all studies report antimicrobial activity parameters such as MIC, MBC, MBIC, or MBEC, and clinical studies often rely on surrogate endpoints such as plaque index or gingival index. These significant differences between studies make comparisons of results difficult to perform and limit the ability to analyze the results both quantitatively and qualitatively to provide clear conclusions. Moreover, many experimental studies have used single- or dual-species biofilms, which are far from replicating the complexity of the microbial flora in the oral cavity. This can lead to an overestimation of antimicrobial efficacy relative to real-life clinical scenarios. Data regarding the pharmacokinetics, local bioavailability, and retention of thymol in the oral cavity are limited. Given that it has low water solubility and is rapidly cleared via saliva, these factors may also influence its clinical performance. Due to this lack of information, it is difficult to optimize the appropriate dose, duration of action, and long-term efficacy. Taking all these factors into account, although thymol exhibits antimicrobial, anti-inflammatory, and antibiofilm effects, the available data do not allow for definitive conclusions regarding its action as a standalone compound (particularly in endodontics, prosthodontics, and in dental materials and nanoformulations, where no clinical data exist).

## 10. Challenges and Future Perspectives

Thymol will have to overcome a few challenges before it can be further optimized for clinical use and expanded into oral-care products. Therefore, the development and clinical validation of standard formulations with good stability, retention time, and bioavailability should be performed. Future research should therefore be directed towards delivery systems that ensure sustained release and enhanced biofilm penetration, while preserving biocompatibility. Thus, the lack of standardized protocols for thymol concentration, formulation composition, frequency of application, duration of exposure, and outcome assessment also represents a barrier to its clinical implementation. There is a lot of heterogeneity in the methodologies being used in studies right now, so it is difficult to make comparisons. Therefore, a future step in research could be the prioritization of standardization of testing protocols and clinically relevant outcome measures that can help in regulatory evaluation and clinical decision-making. Another issue that could be addressed is enriching the data on the pharmacokinetics and pharmacodynamics of thymol. Its antimicrobial activity has been well studied under laboratory conditions, but little is known about its retention in the oral cavity, its penetration into oral tissues and biofilms, its metabolism and its persistence under dynamic clinical conditions. Another important factor to consider is the acceptability of the medication to the patients. Taste perception can have a significant impact on long-term treatment adherence, particularly because thymol has a pungent odor; therefore, organoleptic properties and patient compliance need to be more carefully considered. Although it is recognized as generally recognized as safe (GRAS) by the FDA and has a relatively low toxicological risk profile, more research is needed to determine the therapeutic range, long-term safety, allergenic potential, and optimal dose.

There are numerous opportunities to expand the applications of thymol in modern dentistry, especially given the growing interest in natural compounds; one of the main reasons for this is concern regarding antimicrobial resistance and the adverse reactions caused by the prolonged use of conventional antiseptics. One promising area for future research is the development of up-to-date drug delivery technologies, such as nanosystems that could provide sustained release of thymol to maximize its therapeutic potential while also reducing the frequency of applications. Therefore, future studies should evaluate the long-term stability, release kinetics, biodegradation behavior, and clinical performance of these systems under realistic oral conditions. In addition, the use of thymol in restorative materials, implant coatings, tissue engineering scaffolds, and regenerative therapies could broaden its applications from infection control to preventive and therapeutic biomaterials. Personalized dental therapies represent a promising avenue for the next generation of thymol-based applications. Since oral microbial communities are highly individual, thymol-based therapies might eventually be adapted to each patient’s individual microbiological, inflammatory, or clinical profiles. These approaches may aid in a more targeted and effective management of dental problems. To do so, it is mandatory to assess extended analysis on clinical parameters (e.g., plaque and gingival indices, probing depth, clinical attachment level, clinical and radiographic success rates, postoperative pain, microbial outcomes, and inflammatory biomarkers) to, in fact, confirm its clinical reliability.

In conclusion, future research should be directed toward a stepwise translational pathway including standardized in vitro and ex vivo testing with clinically relevant multispecies biofilms, pharmacokinetic and dose–response studies under dynamic oral conditions, long-term biocompatibility and material compatibility assessments, and well-designed randomized clinical trials comparing thymol-based formulations to current standards of care. Collectively, these advances could pave the way for thymol to evolve from a promising natural bioactive compound into an evidence-based adjunctive therapeutic option in dentistry.

## 11. Conclusions

The current evidence supports thymol as a promising adjunct ingredient of oral-care formulations, especially for preventive and periodontal applications, but not as a stand-alone therapeutic agent. While preliminary and early clinical data are promising, well-designed randomized clinical trials are required to prove its long-term efficacy, optimal formulations and role in routine dental practice.

## Figures and Tables

**Table 1:** Applications of thymol in preventive dentistry.

Formulation	Composition	Antimicrobial Activity Parameters	Reported Outcomes	Targeted Microorganisms	Study Type	Duration, Sample Size	Adverse Effects	Ref.
Mouthwash	Thyme-based	-	Reduced PI and VSC levels, with improvement in oral malodor after 1 week	-	RCT	*n* = 60	-	[58]
Varnish (Cervitec^®^ Plus, Ivoclar Vivadent AG, Schaan, Liechtenstein)	CHX (1%) + THY (1%)	-	Reduced PI, GI, and BI; improved gingival health; reduced *S. mutans* and *P. gingivalis* levels	*S. mutans*, *P. gingivalis*	In vivo	3 months; *n* = 30 patients	No staining or taste alteration; good tolerance	[59]
Varnish (Cervitec^®^, Ivoclar Vivadent AG, Schaan, Liechtenstein)	CHX (1%) + THY (1%)	-	No significant reduction in plaque or gingivitis with 3-monthly application; only a modest effect on GI over time in institutionalized elderly patients	-	RCT	6 months; *n* = 56;	-	[50]
Mouthwash	Sorbitol, propylene glycol, sodium lauryl sulfate, poloxamer 407, benzoic acid, sodium fluoride, eucalyptol, zinc chloride, methyl salicylate, THY, sodium saccharin, sodium benzoate, menthol, aroma, benzyl alcohol, sucralose, green coloring agents	-	Reduced PI and GI after 7 days; decreased the proportion of teeth with PI grades 2–3 by 37%; significantly improved mean plaque scores from baseline; microbiota shifts included increased pathogenic taxa	Mixed oral biofilm; *P. intermedia*, *T. denticola*, *F. nucleatum* subsp. *animalis*	RCT	7 days; *n* = 50	One participant developed mouth ulcers after 3 days	[60]
Varnish (Cervitec^®^ Plus, Ivoclar Vivadent AG, Schaan, Liechtenstein)	CHX (1%) + THY (1%)	-	Significantly reduced salivary bacterial counts over 1, 4, and 12 weeks; sustained antimicrobial effect; had comparable efficacy to fluoride varnish, suggesting potential for caries prevention through suppression of cariogenic microbiota	*S. mutans*	RCT	12 weeks, *n* = 60	Lower acceptability due to bitter taste	[61]
Varnish (Cervitec^®^, Ivoclar Vivadent AG, Schaan, Liechtenstein)	CHX (1%) + THY (1%)	-	Reduced the incidence of root caries and progression of existing lesions (significantly lower increase in height and width); improved lesion texture and color; overall slower lesion growth over 12 months	*S. mutans*; *Lactobacillus* spp.	RCT	12 months; *n* = 68	No staining or other side effects observed; one placebo subject reported a bitter taste	[50]

Abbreviations: THY, thymol; CHX, chlorhexidine; PI, plaque index; GI, gingival index; BI, bleeding index; VSC, volatile sulfur compounds; RCT, randomized controlled trial.

**Table 2:** Applications of thymol in periodontal disease.

Formulation	Composition	Antimicrobial Activity Parameters	Reported Outcomes	Targeted Microorganisms	Study Type	Duration, Sample Size	Adverse Effects	Ref.
Mouthwash	5% alcoholic extract of *Thymus vulgaris*	-	Significant reduction in PI, GI, and salivary IL-6; comparable efficacy to CHX	-	RCT	21 days; *n* = 45 patients	-	[75]
Mouthwash	Thyme-based	-	Reduced the GI and PI, improving gingivitis	-	RCT	1 week; *n* = 60 patients	-	[58]
Varnish (Cervitec^®^ Ivoclar Vivadent AG, Schaan, Liechtenstein)	CHX (1%) + THY (1%)	-	No significant reduction in PI; slight but statistically significant effect over time on the gingival index GI, but no significant difference vs. placebo; limited efficacy in controlling plaque and gingivitis	-	RCT	6 months; *n* = 56 patients	-	[50]
Mouthrinse	THY 0.064%, eucalyptol 0.092%, menthol 0.042%, methyl salicylate 0.060% (alcohol-containing vs. alcohol-free)	-	Adjunctive use significantly reduced PI (≈37%), GI (≈27–28%), and BI after 6 months, with no significant difference between alcohol-containing and alcohol-free formulations.	-	RCT	6 months; *n* = 370 patients (348 completed)	-	[53]
Mouthwash	0.2% CHX + 0.020% THY (alcohol-free)	-	Greater reduction in PI and GI compared to CHX alone; similar reduction in BI	-	RCT	14 days, *n* = 60 patients	Increased staining in both groups	[57]
Mouthrinse (LISTERINE^®^, Johnson & Johnson, NJ, USA)	THY (0.064%), eucalyptol (0.092%), menthol (0.042%), and methyl salicylate (0.060%)	-	Adjunctive reduction in plaque and gingivitis beyond brushing/flossing, with short- and long-term improvement in oral hygiene, gingival health, and plaque control, including orthodontic, xerostomia, periodontal maintenance, and special-needs populations	*P. gingivalis*, *S. mutans*, *L. plantarum*	SR	3–12 months; *n* = 20–766 patients	Two xerostomia subjects developed transient asymptomatic whitish mucosal sloughing at day 7, resolving by day 14 without permanent mucosal changes.	[74]
Gel	THY 0.05% + carvacrol 0.05% in gel base (trehalose, hydroxypropylcellulose, polyvinylpyrrolidone, white mint)	-	Adjunctive home-care gel after scaling and root planing; slight reduction in periodontal bacterial load, but no statistically significant microbiological improvement after treatment	*P. gingivalis*, *T. forsythia*, *T. denticola*, *A. actinomycetemcomitans*, *F. nucleatum*, *C. rectus*	Pilot study	15 days; *n* = 5 patients	No adverse effects reported	[76]

Abbreviations: THY, thymol; CHX, chlorhexidine; PI, plaque index; GI, gingival index; BI, bleeding index; SR, systematic review; RCT, randomized controlled trial.

**Table 3:** Applications of thymol in endodontics.

Formulation	Composition	Antimicrobial Activity Parameters	Reported Outcomes	Targeted Microorganisms	Study Type	Duration, Sample Size	Adverse Effects	Ref.
Solution (Cresophene^®^, Septodont, Saint-Maur-des-Fossés, France	Dexamethasone acetate + THY	-	Reduced intracanal bacterial load comparable to 20% CHX gel; bacterial reduction of 89.85% after 48 h	*E. faecalis*	In vitro	48 h; *n* = 80 extracted single-rooted human teeth	-	[78]
Liquid THY/THY vapor	THY tested in liquid (10–100 mg/mL) and vapor form (1.0–5.0 mg/mL)	MIC: 0.8–1.0 mg/mL (planktonic cultures)	Significant reduction in bacterial viability in early-stage biofilm models; 1.0 mg/mL THY vapor combined with mechanical instrumentation enhanced early biofilm removal, particularly against *E. faecalis*; lower cytotoxicity and no significant induction of pro-inflammatory cytokine gene expression in L-929 cells under low-dose vapor exposure	*E. faecalis*, *S. mutans*, *A. actinomycetemcomitans*	In vitro	-	Direct exposure to liquid THY and CHX caused marked cytotoxicity, whereas 1.0 mg/mL THY vapor showed lower cytotoxicity in L-929 fibroblasts	[77]
Pure compound	THY	-	Significant reduction in CFU counts in mature biofilm after 30–60 min exposure; antimicrobial effect comparable to *Lippia sidoides* essential oil	*E. faecalis* isolated from infected root canals	In vitro	72 h	-	[83]
Oil	Thyme oil containing THY (70.76%)	MIC: 512 μg/mL	Inhibited biofilm formation at sub-MIC concentrations (128–256 μg/mL); reduced cell motility and EPS synthesis; downregulated *ebp* and *epa* biofilm-related genes; at 2048–4096 μg/mL reduced viable counts in mature 3-day biofilms after 30 min	*E. faecalis*	In vitro	3-day biofilm model; biofilm assessed at 12, 24, 48, and 72 h; mature biofilms exposed for 5, 15, 30, or 60 min;	-	[84]

Abbreviations: THY, thymol; CHX, chlorhexidine; MIC, minimum inhibitory concentration; EPS, exopolysaccharide; CFU, colony-forming units.

**Table 4:** Applications of thymol in Prosthodontics and Prosthesis-Associated Candidiasis.

Formulation	Composition	Antimicrobial Activity Parameters	Reported Outcomes	Targeted Microorganisms	Study Type	Duration, Sample Size	Adverse Effects	Ref.
Denture cleanser	Denture-cleansing tablet containing THY; 1 tablet dissolved in 150 mL sterile distilled water	-	In planktonic time-kill assays, THY cleanser showed >99.9% killing of *C. albicans* after 5 min. In 72-h multispecies biofilms on acrylic resin, longer immersion was required; 3–6 h exposure produced antibiofilm effects comparable to 10 min of 0.5% NaClO. The cleanser also showed stain-removal activity comparable to Polident^®^ denture cleanser	*C. albicans*; multispecies biofilm also included *S. mutans*, *S. sanguinis*, *S. aureus*, and *E. coli*	In vitro	Time-kill: 5, 15, 30 min, 1 h, and 3 h; biofilm model: 72-h multispecies biofilm; biofilm immersion: 30 min, 3 h, and 6 h; material and cytotoxicity testing simulated 6 months of daily cleansing	No cytotoxicity in L929 fibroblasts or significant changes in acrylic resin roughness or color after simulated 6-month daily immersion.	[92]
Pure compound	THY	MIC: 39 μg/mL for *C. albicans* and *C. krusei*; 78 μg/mL for *C. tropicalis*. MFC values MFC/MIC < 4 for all tested strains	Fungicidal activity against oral Candida species; exogenous ergosterol increased thymol MIC against C. albicans from 39 to 312.5 μg/mL, while combination with nystatin reduced both MICs by 87.4% (FIC index 0.25).	*C. albicans*, *C. tropicalis*, *C. krusei*	In vitro	MIC determined after 24 h; MFC after 48 h; mechanistic and synergism assays over 48 h; sorbitol assay evaluated at 7 day	-	[34]
Solution	THY	MIC: 125–150 μg/mL against *C. albicans* strains	Inhibited hyphal formation and viability, reducing hyphal forms from ~94% in controls to 14.3% at MIC after 6 h, with membrane damage and reduced viability.	*C. albicans*	In vitro	6 h incubation; *n* = 3 strains (2 clinical isolates + 1 ATCC strain)	-	[87]
Solution	THY	MIC: 125 μg/mL for both *C. albicans* strains	Inhibited early biofilm formation and disrupted mature biofilms, reducing metabolic activity by >90% at 2× MIC after 24 h, with disrupted biofilm architecture and reduced filamentous structures.	*C. albicans*	In vitro	Biofilm exposure for 6, 12, and 24 h; *n* = 2 strains of *C. albicans* (ATCC 3153A and ATCC MYA 2876)	-	[91]
Pure compound	THY alone; piperine + THY	MBI for THY: 32 µg/mL for *C. albicans* ATCC 90028; 32–128 µg/mL for clinical isolates. Synergistic antibiofilm combinations of piperine + THY: 8 + 8, 8 + 4, 8 + 2, and 4 + 8 µg/mL; FICI ≤ 0.5	Thymol inhibited >87–90% of biofilm formation at MBIC; piperine + THY showed synergistic antibiofilm effects, reducing adhered cells by >2 log, hyphal elongation, phenotypic switching, and related gene expression.	*C. albicans* ATCC 90028 and four clinical isolates	In vitro	Biofilm assays: 48 h incubation; antihyphal assay: 5–7 days on solid medium; yeast-to-hyphal assay: 4 h; hyphal-to-yeast assay: 2 h after 4 h hyphal induction	No haemolytic activity on human erythrocytes and no morphological toxicity on human buccal epithelial cells at tested concentrations	[90]
Pure compound	THY	MIC: 500 μM for *S. oralis*, *A. naeslundii*, *V. parvula*, *F. nucleatum*, *A. actinomycetemcomitans*, and *C. albicans*; 1 mM for *P. gingivalis*. Minimum concentration inhibiting *C. albicans* filamentation: 250 μM	Inhibited C. albicans filamentous growth, reduced F. nucleatum and P. gingivalis viability, partially reversed dysbiotic biofilm structure, and increased biofilm roughness with reduced compact architecture.	*C. albicans* SC5314 within multispecies peri-implant biofilm, including *S. oralis*, *A. naeslundii*, *V. parvula*, *F. nucleatum*, *P. gingivalis*, and *A. actinomycetemcomitans*	In vitro	Biofilms developed for 72 h on implant surfaces; filamentation assay: 48 h; MIC testing: 24 h incubation plus 72 h plating confirmation	-	[14]
Pure compound	THY	MIC_50_: 16 µg/mL; BIC_50_: 32 µg/mL; BEC_50_: 128 µg/mL	THY showed antifungal and antibiofilm activity against *C. tropicalis*. It inhibited planktonic growth, impaired biofilm formation, and affected mature biofilms. THY also increased ROS production	*C. tropicalis*	In vitro	MIC assay: 48 h; biofilm inhibition: 48 h; preformed biofilm treatment: 24 h biofilm + 24 h treatment	-	[93]
Denture cleanser	Sodium bicarbonate (2.38 g) + THY oil (1.24 g) in 100 mL distilled water	-	Demonstrated the lowest fungal adhesion among tested cleansers; significantly reduced *Candida* colonization on Co–Cr denture base and lowered surface roughness	*C. albicans*	In vitro	Immersion for 1 month using 0.5 h/day and 8 h/day regimens; *n* = 36 Co–Cr denture base specimens; 6 samples per cleanser group	No adverse effects reported; reduced roughness compared with control	[94]

Abbreviations: THY, thymol; MIC, minimum inhibitory concentration; MFC, minimum fungicidal concentration; MBIC, minimum biofilm inhibitory concentration; FIC, fractional inhibitory concentration; FICI, fractional inhibitory concentration index; BIC_50_, biofilm inhibitory concentration required to inhibit 50% of biofilm formation; BEC_50_, biofilm eradication concentration required to eradicate 50% of established biofilm; ROS, reactive oxygen species.

**Table 5:** Applications of thymol in Dental Materials and Drug Delivery Systems.

Formulation	Composition	Antimicrobial Activity Parameters	Reported Outcomes	Targeted Microorganism	Study Type	Duration, Sample Size	Adverse Effects	Ref.
UiO-66 metal–organic framework	THY loaded into zirconium-based UiO-66 metal–organic framework	MBC: 0.313 mg/mL (*C. albicans*, *E. coli*), 1.25 mg/mL (*S. aureus*)	It presented excellent biocompatibility (≥50% cell viability); in vivo studies also confirmed Thy@UiO-66 decreased inflammation, while stimulating the formation of bone	*C. albicans*, *E. coli*, and *S. aureus*	In vitro and in vivo	-	No significant cytotoxicity; >50% cell viability at 1.25 mg/mL	[100]
THY-loaded microsponge in situ gel	THY	-	Reduced gingival inflammation, tooth mobility, and alveolar bone destruction; decreased inflammatory biomarkers; prevented osteoclastogenesis and osteoblast apoptosis	*S. mutans*, *C. albicans*	In vitro and in vivo	-	-	[99]
Nanoparticles	Clove oil + THY nanoencapsulated in chitosan/poly-γ-glutamic acid nanoparticles	MIC: 0.5 mg/mL (both agents); FIC ≤ 0.5 (synergistic after nanoencapsulation); >2 log_10_ CFU reduction in time-kill assay	Enhanced and prolonged antimicrobial activity due to synergistic effect; significantly reduced salivary bacterial load and maintained activity in the oral cavity compared with free compounds	*S. mutans*, *S. sobrinus*	In vitro and in vivo	Time-kill: up to 48 h; mouth-rinse: 30–90 min; *n* = 18 healthy volunteers	-	[96]
THY–chitosan systems	THY incorporated into chitosan-based systems, including nanogels, nanoparticles, micelles, films, hydrogels, and nanocomposites	THY-loaded chitosan nanogels reduced MIC values 4–6-fold compared with free THY	THY chitosan systems improved antimicrobial, antibiofilm, anti-inflammatory, antioxidant, wound-healing, and regenerative potential compared with free thymol in several included studies	*S. aureus*, *S. mutans*, *A. baumannii*, *P. aeruginosa*, *E. coli*, *C. albicans*	SR	-	-	[19]
Nanoemulsions	THY incorporated into lecithin/Pluronic^®^ P123 nanoemulsions with grape seed oil	-	Improved THY stability, local buccal distribution, antioxidant activity, and sustained release, supporting potential use in oral infections	-	In vitro and ex vivo	Stability (45 days); in vitro release (72 h); ex vivo buccal permeability (7 h); antioxidant assay (48 h)	-	[13]
Nanogel	Poly (acrylamide) nanogel loaded with THY	-	Boosted antifungal activity with increased zone of inhibition vs. control; improved permeability and sustained drug release (75.47–99.62%)	*C. albicans*	In vitro and ex vivo	Drug release: 24 h; ex vivo permeation: 12 h; antifungal assay: 24 h and 120 h	-	[103]

Abbreviations: THY, thymol; MBC, minimum bactericidal concentration; MIC, minimum inhibitory concentration; FIC, fractional inhibitory concentration; CFU, colony-forming unit; SR, systematic review.

## Data Availability

No new data were created or analyzed in this study. Data sharing is not applicable to this article.

## References

[1] Oral Health. https://www.who.int/health-topics/oral-health#tab=tab_1.

[2] Mehta V., Mathur A., Tripathy S., Rizwan S.A., Sharma T. (2024). Effectiveness of herbal oral care products in reducing dental plaque and gingivitis: An overview of systematic reviews. Can. J. Dent. Hyg..

[3] Li L., Zhang Q., Yang D., Yang S., Zhao Y., Jiang M., Wang X., Zhao L., Liu Q., Lu Z. (2023). Tooth loss and the risk of cognitive decline and dementia: A meta-analysis of cohort studies. Front. Neurol..

[4] Priya A., Selvaraj A., Divya D., Raja R.K., Pandian S.K. (2021). In vitro and in vivo anti-infective potential of thymol against early childhood caries causing dual species *Candida albicans* and *Streptococcus mutans*. Front. Pharmacol..

[5] Jayakumar S., Sridhar D., John B.M., Arumugam K., Ponnusamy P., Pulidindi H. (2024). Biofilm in endodontic infection and its advanced therapeutic options—An updated review. J. Pharm. Bioallied Sci..

[6] Li Y., Xing Z., Wang S., Wang Y., Wang Z., Dong L. (2023). Disruption of biofilms in periodontal disease through the induction of phase transition by cationic dextrans. Acta Biomater..

[7] Qiu J., Roza M.P., Colli K.G., Dalben Y.R., Maifrede S.B., Valiatti T.B., Novo V.M., Cayô R., Grão-Velloso T.R., Gonçalves S.S. (2023). Candida-associated denture stomatitis: Clinical, epidemiological, and microbiological features. Braz. J. Microbiol..

[8] Liu H.Y., Prentice E.L., Webber M.A. (2024). Mechanisms of antimicrobial resistance in biofilms. npj Antimicrob. Resist..

[9] Abbood H.M., Hijazi K., Gould I.M. (2023). Chlorhexidine resistance or cross-resistance, that is the question. Antibiotics.

[10] Dumitrel S.-I., Matichescu A., Dinu S., Buzatu R., Popovici R., Dinu D.C., Bratu D.C. (2024). New insights regarding the use of relevant synthetic compounds in dentistry. Molecules.

[11] Kowalczyk A., Przychodna M., Sopata S., Bodalska A., Fecka I. (2020). Thymol and thyme essential oil—New insights into selected therapeutic applications. Molecules.

[12] Nica D.F., Cosoroabă R.M., Dinu Ș., Dumitrel Ș.-I., Chioran D., Tănase A., Popa M. (2026). Exploring thymol’s cytocompatibility and potential selective cytotoxicity in human primary gingival fibroblasts and pharyngeal carcinoma cells: An in vitro and in ovo investigation. Dent. J..

[13] Saatkamp R.H., Sanches M.P., Gambin J.P.D., Amaral B.R., Farias N.S., Caon T., Müller C.M.O., Parize A.L. (2023). Development of thymol nanoemulsions with potential application in oral infections. J. Drug Deliv. Sci. Technol..

[14] Bravo E., Arce M., Herrera D., Sanz M. (2024). The effect of xanthohumol and thymol on *Candida albicans* filamentation and its impact on the structure, size, and cell viability of biofilms developed over implant surfaces. Cells.

[15] van Swaaij B.W.M., Van der Weijden G.A., Smith R.J., Timmerman M.F., Slot D.E. (2024). Essential oils mouthwash with or without alcohol in relation to effect on parameters of plaque and gingivitis: A systematic review and meta-analysis. Int. J. Dent. Hyg..

[16] Milić M., Bolanča I., Gjirlić D., Benković V. (2019). Assessment of Listerine Cool Mint mouthwash influence on possible DNA damage measured by buccal micronucleus cytome assay—Preliminary results. Genet. Appl..

[17] Gonzalez A., Miñán A.G., Grillo C.A., Prieto E.D., Schilardi P.L., Fernández Lorenzo de Mele M.A. (2020). Characterization and antimicrobial effect of a bioinspired thymol coating formed on titanium surface by one-step immersion treatment. Dent. Mater..

[18] Najafloo R., Behyari M., Imani R., Nour S. (2020). A mini-review of thymol incorporated materials: Applications in antibacterial wound dressing. J. Drug Deliv. Sci. Technol..

[19] Kowalczyk A., Twarowski B., Fecka I., Tuberoso C.I.G., Jerković I. (2024). Thymol as a component of chitosan systems—Several new applications in medicine: A comprehensive review. Plants.

[20] Thymol. https://pubchem.ncbi.nlm.nih.gov/compound/Thymol.

[21] Hikal W., Tkachenko K., Said-Al Ahl H.A.H., Sany H., Sabra A.S., Baeshen R., Bratovcic A. (2021). Chemical composition and biological significance of thymol as antiparasitic. Open J. Ecol..

[22] Wadhawan R., Singla P., Mishra S., Mansuri S., Kumar S., Raj N., Devi L.M. (2024). Role of wonder drug thyme in dentistry: A review. J. Orofac. Health Sci..

[23] Liu S., Lei J., Liu H., Chen T., Gao C., Luo D., Liao Q., Liu X., Dong P. (2026). Thymol: Properties, synthesis, mechanism of action, and applications. Front. Nutr..

[24] Escobar A., Pérez M., Romanelli G., Blustein G. (2020). Thymol bioactivity: A review focusing on practical applications. Arab. J. Chem..

[25] Khwaza V., Aderibigbe B.A. (2025). Antibacterial activity of selected essential oil components and their derivatives: A review. Antibiotics.

[26] Li Q., Huang K.X., Pan S., Su C., Bi J., Lu X. (2022). Thymol disrupts cell homeostasis and inhibits the growth of *Staphylococcus aureus*. Contrast Media Mol. Imaging.

[27] Barciela P., Perez-Vazquez A., Jorge A.O.S., Pereira A.G., Oliveira M.B.P.P., Carpena M., Prieto M.A., Jafari S.M., Darvishi Harzevili F., Karaca A.C. (2025). Microbial Production of Thymol. Microbial Production of Food Bioactive Compounds.

[28] Liang C., Huang S., Geng Y., Huang X., Chen D., Lai W., Guo H., Deng H., Fang J., Yin L. (2022). A study on the antibacterial mechanism of thymol against *Aeromonas hydrophila* in vitro. Aquac. Int..

[29] Yin L., Liang C., Wei W., Huang S., Ren Y., Geng Y., Huang X., Chen D., Guo H., Fang J. (2022). The antibacterial activity of thymol against drug-resistant *Streptococcus iniae* and its protective effect on channel catfish (*Ictalurus punctatus*). Front. Microbiol..

[30] Jung K.-W., Chung M.-S., Bai H.-W., Chung B.-Y., Lee S. (2021). Investigation of antifungal mechanisms of thymol in the human fungal pathogen, *Cryptococcus neoformans*. Molecules.

[31] Kmoch M., Loubová V., Švecová R., Jílková B. (2025). Effect of essential oil components on the growth inhibition of *Fusarium solani* var. *coeruleum* during potato storage. Agronomy.

[32] Ramadhan M.H., Abdul-Ameer F. (2025). Antifungal efficacy of thymol powder addition on *Candida albicans* adhesion to room temperature vulcanized maxillofacial silicone: An in vitro study. Front. Biomed. Technol..

[33] Zhang J., Ren F., Gao H., Li Y., Yin X., Zhou Z., Zhang Z., Qiu H., Cai Y., Shcherbakova L. (2026). Antifungal activity and action mechanism of thymol, a mycotoxin inhibitor, against *Fusarium asiaticum*. Food Chem..

[34] de Castro R.D., de Souza T.M.P.A., Bezerra L.M.D., Ferreira G.L.S., Costa E.M.M.B., Cavalcanti A.L. (2015). Antifungal activity and mode of action of thymol and its synergism with nystatin against *Candida* species involved with infections in the oral cavity: An in vitro study. BMC Complement. Altern. Med..

[35] Utami D.T., Pratiwi S.U.T., Haniastuti T., Hertiani T. (2021). Eugenol and thymol as potential inhibitors for polymicrobial oral biofilms: An in vitro study. J. Int. Oral Health.

[36] Valliammai A., Selvaraj A., Yuvashree U., Aravindraja C., Pandian S.K. (2020). *sarA*-dependent antibiofilm activity of thymol enhances the antibacterial efficacy of rifampicin against *Staphylococcus aureus*. Front. Microbiol..

[37] Yuan Z., Dai Y., Ouyang P., Rehman T., Hussain S., Zhang T., Yin Z., Fu H., Lin J., He C. (2020). Thymol inhibits biofilm formation, eliminates pre-existing biofilms, and enhances clearance of methicillin-resistant *Staphylococcus aureus* (MRSA) in a mouse peritoneal implant infection model. Microorganisms.

[38] Gabbai-Armelin P.R., Sales L.S., Ferrisse T.M., De Oliveira A.B., De Oliveira J.R., Giro E.M.A., Brighenti F.L. (2022). A systematic review and meta-analysis of the effect of thymol as an anti-inflammatory and wound healing agent. Phytother. Res..

[39] Gago C., Serralheiro A., Miguel M.G. (2025). Anti-inflammatory activity of thymol and thymol-rich essential oils: Mechanisms, applications, and recent findings. Molecules.

[40] Herrera-Bravo J., Belén L.H., Reyes M.E., Silva V., Fuentealba S., Paz C., Loren P., Salazar L.A., Sharifi-Rad J., Calina D. (2024). Thymol as adjuvant in oncology: Molecular mechanisms, therapeutic potentials, and prospects for integration in cancer management. Naunyn Schmiedebergs Arch. Pharmacol..

[41] Alamri M.A., Abdel-Kader M.S., Salkini M.A., Alamri M.A. (2024). Thymol and carvacrol derivatives as anticancer agents; synthesis, in vitro activity, and computational analysis of biological targets. RSC Adv..

[42] Faur A., Todor L., Dinu S., Porumb A., Todor S.A., Talpos-Niculescu I.C., Cosoroabă R.M., Popovici R.A., Olariu I. (2022). Applications of thyme extracts on diseases of the oral cavity. Med. Evol..

[43] De La Chapa J.J., Singha P.K., Lee D.R., Gonzales C.B. (2018). Thymol inhibits oral squamous cell carcinoma growth via mitochondria-mediated apoptosis. J. Oral Pathol. Med..

[44] Pouyamanesh G., Ameli N., Metanat Y., Khorrami A., Abbasinezhad-Moud F., Seraj F.Q.M., Ferns G.A., Bahrami A. (2025). Thymol enhances 5-fluorouracil cytotoxicity by reducing migration and increasing apoptosis and cell cycle arrest in esophageal cancer cells: An in-vitro study. Indian J. Clin. Biochem..

[45] Sampaio L.A., Pina L.T.S., Serafini M.R., Tavares D.S., Guimarães A.G. (2021). Antitumor effects of carvacrol and thymol: A systematic review. Front. Pharmacol..

[46] Marinković J., Rakašević D., Nemoda M., Nikolić B., Marković T., Matijević S., Marković D. (2023). EO-based mouthwashes: Is there something that should be known?. Balk. J. Dent. Med..

[47] Song S.R., Moon K., Oh K., Ha W. (2021). In-vitro and in-vivo characteristics of EUTHYMOL^®^ mouthwash on fluoride retention. Int. J. Clin. Prev. Dent..

[48] Kim H., Choi W., Lee W., Kim W., Oh K., Ha W. (2021). Inhibitory effect of EUTHYMOL original toothpaste on VSC-producing oral bacteria. Int. J. Clin. Prev. Dent..

[49] VivaDent Protecting Family. https://www.ivoclar.com/en_li/products/prevention-and-care/vivadent-protecting-family.

[50] Clavero J., Baca P., González M.P., Valderrama M.J. (2006). Efficacy of chlorhexidine-thymol varnish (Cervitec) against plaque accumulation and gingival inflammation in a geriatric population. Gerodontology.

[51] Nikfallah A., Mohammadi A., Ahmadakhondi M., Ansari M. (2023). Synthesis and physicochemical characterization of mesoporous hydroxyapatite and its application in toothpaste formulation. Heliyon.

[52] Miladi H., Zmantar T., Kouidhi B., Al Qurashi Y.M.A., Bakhrouf A., Chaabouni Y., Mahdouani K., Chaieb K. (2017). Synergistic effect of eugenol, carvacrol, thymol, *p*-cymene and γ-terpinene on inhibition of drug resistance and biofilm formation of oral bacteria. Microb. Pathog..

[53] Lynch M.C., Cortelli S.C., McGuire J.A., Zhang J., Ricci-Nittel D., Mordas C.J., Aquino D.R., Cortelli J.R. (2018). The effects of essential oil mouthrinses with or without alcohol on plaque and gingivitis: A randomized controlled clinical study. BMC Oral Health.

[54] Kashi M., Varseh M., Hariri Y., Chegini Z., Shariati A. (2025). Natural compounds: New therapeutic approach for inhibition of *Streptococcus mutans* and dental caries. Front. Pharmacol..

[55] Motallaei M.N., Yazdanian M., Tebyanian H., Tahmasebi E., Alam M., Abbasi K., Seifalian A., Ranjbar R., Yazdanian A. (2021). The current strategies in controlling oral diseases by herbal and chemical materials. Evid.-Based Complement. Altern. Med..

[56] Tzimas K., Antoniadou M., Varzakas T., Voidarou C. (2024). Plant-derived compounds: A promising tool for dental caries prevention. Curr. Issues Mol. Biol..

[57] Amoian B., Omidbakhsh M., Khafri S. (2017). The clinical evaluation of Vi-one chlorhexidine mouthwash on plaque-induced gingivitis: A double-blind randomized clinical trial. Electron. Physician.

[58] Altındal D., Deveci K.C., Öner Talmaç A.G., Talmaç A.C., Çalışır M. (2024). Effects of thyme on halitosis in gingivitis patients: Can thyme mouthwash prevent halitosis—A randomized trial. Int. J. Dent. Hyg..

[59] George A.M., Kalangi S.K., Vasudevan M., Krishnaswamy N.R. (2010). Chlorhexidine varnishes effectively inhibit *Porphyromonas gingivalis* and *Streptococcus mutans*—An in vivo study. J. Indian Soc. Periodontol..

[60] Dutot M., Le Besco M., Mathurin O., Fong S.-B., Meuric V., Tanter C. (2026). Control of dental plaque and gingival inflammation by natural ingredients-based mouthwash. Dent. J..

[61] Ben Khadra G.M., Arrag E.A., Alammori M., AlKadi M.F. (2019). The effect of chlorhexidine-thymol and fluoride varnishes on the levels of *Streptococcus mutans* in saliva in children aged 6–8 years. Indian J. Dent. Res..

[62] Botelho M.A., Nogueira N.A.P., Bastos G.M., Fonseca S.G.C., Lemos T.L.G., Matos F.J.A., Montenegro D., Heukelbach J., Rao V.S., Brito G.A.C. (2007). Antimicrobial activity of the essential oil from *Lippia sidoides*, carvacrol and thymol against oral pathogens. Braz. J. Med. Biol. Res..

[63] Vlachojannis C., Chrubasik-Hausmann S., Hellwig E., Al-Ahmad A. (2015). A preliminary investigation on the antimicrobial activity of Listerine^®^, its components, and of mixtures thereof. Phytother. Res..

[64] Park S.-Y., Raka R.N., Hui X.-L., Song Y., Sun J.-L., Xiang J., Wang J., Jin J.-M., Li X.-K., Xiao J.-S. (2023). Six Spain *Thymus* essential oils composition analysis and their in vitro and in silico study against *Streptococcus mutans*. BMC Complement. Med. Ther..

[65] Shrestha A., Rimal J., Rao A., Sequeira P.S., Doshi D., Bhat G.K. (2011). In vitro antifungal effect of mouth rinses containing chlorhexidine and thymol. J. Dent. Sci..

[66] Almudahi A. (2022). Enhancing Root Caries Lesion Prevention by Combining Two American Dental Association-Recommended Preventive Agents. Master’s Thesis.

[67] Rani N., Singla R.K., Narwal S., Tanushree, Kumar N., Rahman M.M. (2022). Medicinal plants used as an alternative to treat gingivitis and periodontitis. Evid.-Based Complement. Altern. Med..

[68] Richa G., Pudakalkatti P.S., Joshi V. (2017). Evaluation and comparison of the antimicrobial effect of two different mouthwashes on selected periodontal pathogens: An in vitro study. J. Curr. Res. Sci. Med..

[69] Gusmão I.C.C.P., Pithon M.M., Santos A.E., Silva T.S., Sampaio F.C. (2022). Antibacterial activity of *Lippia sidoides* Cham against periodontopathogens: An in vitro study. Res. Soc. Dev..

[70] Hashim N.T., Babiker R., Chaitanya N.C.S.K., Mohammed R., Priya S.P., Padmanabhan V., Ahmed A., Dasnadi S.P., Islam M.S., Gismalla B.G. (2025). New insights in natural bioactive compounds for periodontal disease: Advanced molecular mechanisms and therapeutic potential. Molecules.

[71] Guo J., Gao M., Niu H., Liu D., Chen X. (2025). Thymol inhibits LPS-induced inflammation in rat gingival fibroblasts by regulating inflammatory pathways. J. Oral Sci. Res..

[72] Mehrotra N., Singh S. (2023). Periodontitis. StatPearls.

[73] Sapkota M., Li L., Kim S.-W., Soh Y. (2018). Thymol inhibits RANKL-induced osteoclastogenesis in RAW264.7 and BMM cells and LPS-induced bone loss in mice. Food Chem. Toxicol..

[74] Alshehri F.A. (2018). The use of mouthwash containing essential oils (LISTERINE^®^) to improve oral health: A systematic review. Saudi Dent. J..

[75] Ramadhan L.I., Rashid S.K. (2023). Clinical and anti-inflammatory effects of *Thymus* extract mouthwash in patients with gingivitis. J. Univ. Duhok.

[76] Lauritano D., Pazzi D., Iapichino A., Gaudio R.M., Di Muzio M., Lo Russo L., Pezzetti F. (2016). Evaluation of the efficacy of a new oral gel containing carvacrol and thymol for home oral care in the management of chronic periodontitis using PCR analysis: A microbiological pilot study. J. Biol. Regul. Homeost. Agents.

[77] Manuel M.P.D., Shih Y.-H., Hsia S.-M., Wang T.-H., Tseng Y.-H., Tu M.-G., Shieh T.-M. (2026). Evaluating thymol vapor for biofilm removal and biocompatibility in curved root canal models in vitro. J. Dent. Sci..

[78] Gadekar T., Shetty R.M., Yadadi S.S., Adtani P., Desai V.B., Afrashtehfar K.I. (2025). Comparative evaluation of the antimicrobial efficacy of 20% chlorhexidine, 3% sodium hypochlorite, and dexamethasone acetate with thymol as a root canal disinfectant against *Enterococcus faecalis*: An in-vitro feasibility study. Open Dent. J..

[79] Hahn C.-L., Hanford K. (2021). An in vitro model to study the colonization and tubular invasion of *Enterococcus faecalis*. J. Endod..

[80] Elashiry M.M., Bergeron B.E., Tay F.R. (2023). *Enterococcus faecalis* in secondary apical periodontitis: Mechanisms of bacterial survival and disease persistence. Microb. Pathog..

[81] Zong B., Li X., Xu Q., Wang D., Gao P., Zhou Q. (2022). Enhanced eradication of *Enterococcus faecalis* biofilms by quaternized chitosan-coated upconversion nanoparticles for photodynamic therapy in persistent endodontic infections. Front. Microbiol..

[82] Xie Y., Cheng X., Li Y., Xu X. (2024). Research progress on the dentin adhesion of *Enterococcus faecalis* and its influencing factors. J. Prev. Treat. Stomatol. Dis..

[83] Veras H.N.H., Rodrigues F.F.G., Botelho M.A., Menezes I.R.A., Coutinho H.D.M., da Costa J.G.M. (2014). Antimicrobial effect of *Lippia sidoides* and thymol on *Enterococcus faecalis* biofilm of the bacterium isolated from root canals. Sci. World J..

[84] Liu F., Jin P., Gong H., Sun Z., Du L., Wang D. (2020). Antibacterial and antibiofilm activities of thyme oil against foodborne multiple antibiotics-resistant *Enterococcus faecalis*. Poult. Sci..

[85] Bajunaid S.O. (2022). How effective are antimicrobial agents on preventing the adhesion of *Candida albicans* to denture base acrylic resin materials? A systematic review. Polymers.

[86] Salehi B., Mishra A.P., Shukla I., Sharifi-Rad M., Contreras M.d.M., Segura-Carretero A., Fathi H., Nasri Nasrabadi N., Kobarfard F., Sharifi-Rad J. (2018). Thymol, thyme, and other plant sources: Health and potential uses. Phytother. Res..

[87] Braga P.C., Alfieri M., Culici M., Dal Sasso M. (2007). Inhibitory activity of thymol against the formation and viability of *Candida albicans* hyphae. Mycoses.

[88] Jafri H., Ahmad I. (2020). *Thymus vulgaris* essential oil and thymol inhibit biofilms and interact synergistically with antifungal drugs against drug resistant strains of *Candida albicans* and *Candida tropicalis*. J. Mycol. Med..

[89] Kauser S., Raj N., Ahmedi S., Manzoor N. (2024). Mechanistic insight into the membrane disrupting properties of thymol in *Candida* species. Microbe.

[90] Priya A., Nivetha S., Pandian S.K. (2021). Synergistic interaction of piperine and thymol on attenuation of the biofilm formation, hyphal morphogenesis and phenotypic switching in *Candida albicans*. Front. Cell. Infect. Microbiol..

[91] Braga P.C., Culici M., Alfieri M., Dal Sasso M. (2008). Thymol inhibits *Candida albicans* biofilm formation and mature biofilm. Int. J. Antimicrob. Agents.

[92] Kaypetch R., Rudrakanjana P., Tua-Ngam P., Tosrisawatkasem O., Thairat S., Tonput P., Tantivitayakul P. (2023). Effects of two novel denture cleansers on multispecies microbial biofilms, stain removal and the denture surface: An in vitro study. BMC Oral Health.

[93] Chatrath A., Gangwar R., Kumari P., Prasad R. (2019). In vitro anti-biofilm activities of citral and thymol against *Candida tropicalis*. J. Fungi.

[94] Jaffer N.T. (2011). The influence of natural products as denture cleansers on *Candida albicans* colonization to cobalt–chromium alloy denture base material. Al-Rafidain Dent. J..

[95] Bazán T.A.X.N., Silva I.S.S., Carvalho E.M., Maia Filho E.M., Nascimento L.C., Tavarez R.R.D.J. (2026). Effect of *Thymus vulgaris* essential oil as an antifungal agent on the flexural strength, surface roughness, and color stability of polymethyl methacrylate acrylic resin. Sci. Rep..

[96] Lee J.-S., Choi Y.S., Lee H.G. (2020). Synergistic antimicrobial properties of nanoencapsulated clove oil and thymol against oral bacteria. Food Sci. Biotechnol..

[97] Rezaeian Z., Beigi-Boroujeni S., Atai M., Ebrahimibagha M., Özcan M. (2019). A novel thymol-doped enamel bonding system: Physico-mechanical properties, bonding strength, and biological activity. J. Mech. Behav. Biomed. Mater..

[98] Wu L., Tu Y., Xiao S., Zeng J., Sun G., Li Y. (2026). Recent perspectives on precision-targeting therapy against oral biofilm. J. Oral Microbiol..

[99] Patole V.C., Chaudhari S.P. (2021). Development of thymol microsponges loaded in situ gel for the treatment of periodontitis. Curr. Drug Deliv..

[100] Zheng M., Huang Y., Hu W., Li R., Wang J., Han M., Li Z. (2024). Evaluation of the antibacterial, anti-inflammatory, and bone-promoting capacity of UiO-66 loaded with thymol or carvacrol. ACS Appl. Mater. Interfaces.

[101] Lim D.Y., Lee J.-S., Lee H.G. (2023). Nano-encapsulation of a combination of clove oil and thymol and their application in fresh-cut apples and raw minced beef. Food Control.

[102] Valenti C., Federici M.I., Coniglio M., Betti P., Pancrazi G.P., Tulli O., Masciotti F., Nanussi A., Pagano S. (2024). Mechanical and biological properties of polymer materials for oral appliances produced with additive 3D printing and subtractive CAD-CAM techniques compared to conventional methods: A systematic review and meta-analysis. Clin. Oral Investig..

[103] Omar A.M., Abd El-Azem A.M., Abd El-Hamid E.S., El-Khazragy N.N., Fathi R.F. (2023). Effect of nanoformulated thymol, nanoformulated doxorubicin and their combination on oral squamous cell carcinoma cell line: A comparative ex-vivo study. J. Adv. Zool..

[104] Thosar N.R., Chandak M., Bhat M., Basak S. (2018). Evaluation of antimicrobial activity of two endodontic sealers: Zinc oxide with thyme oil and zinc oxide eugenol against root canal microorganisms—An in vitro study. Int. J. Clin. Pediatr. Dent..

[105] Haider S., Abidi Y.A., Ahmad A., Islam F., Batool A., Raza S.S. (2025). Enhanced fluoride release from glass ionomer cements and compomers modified with clove oil and thymol: A step toward advanced caries prevention. Indus J. Biosci. Res..

[106] Alizadeh N., Nazari F. (2022). Thymol essential oil/β-cyclodextrin inclusion complex into chitosan nanoparticles: Improvement of thymol properties in vitro studies. J. Mol. Liq..

[107] Naik J.B., Rajput R.L., Narkhede J.S., Mujumdar A., Patil P.B. (2021). Synthesis and evaluation of UV cross-linked poly(acrylamide)-loaded thymol nanogel for antifungal application in oral candidiasis. J. Polym. Res..

[108] Soo Hoo G.W., Hinds R.L., Dinovo E., Renner S.W. (2003). Fatal large-volume mouthwash ingestion in an adult: A review and the possible role of phenolic compound toxicity. J. Intensive Care Med..

[109] Carl Roth GmbH + Co. KG (2024). Safety Data Sheet: Thymol ≥ 99%.

[110] Lachenmeier D.W., Monakhova Y.B., Markova M., Kuballa T., Rehm J. (2013). What happens if people start drinking mouthwash as surrogate alcohol? A quantitative risk assessment. Food Chem. Toxicol..

[111] Chowdhury R.I., Bappi M.H., Ahammed S., Hossain M.S., Akbor M.S., Emon M., Islam A., Prottay A.A.S., Shahria N., Altemani F.H. (2026). Anti-nociceptive property of thymol: Animal study with molecular docking and MD simulation studies. Silico Pharmacol..

[112] Tariq M., Kiyani A., Ahmed A., Raja Z.S., Hassan U. (2025). Evaluating the analgesic, hemostatic, and antimicrobial potential of a novel herbal formulation for dental use. Cureus.

